# Bioresorbable Nanostructured Chemical Sensor for Monitoring of pH Level In Vivo

**DOI:** 10.1002/advs.202202062

**Published:** 2022-05-26

**Authors:** Martina Corsi, Alessandro Paghi, Stefano Mariani, Giulia Golinelli, Aline Debrassi, Gabriella Egri, Giuseppina Leo, Eleonora Vandini, Antonietta Vilella, Lars Dähne, Daniela Giuliani, Giuseppe Barillaro

**Affiliations:** ^1^ Dipartimento di Ingegneria dell'Informazione Università di Pisa via G. Caruso 16 Pisa 56122 Italy; ^2^ Department of Medical and Surgical Sciences for Children & Adults University‐Hospital of Modena and Reggio Emilia Via del Pozzo 71 Modena 41124 Italy; ^3^ Surflay Nanotec GmbH Max‐Planck‐Straße 3 12489 Berlin Germany; ^4^ Department of Biomedical Metabolic and Neural Sciences University of Modena and Reggio Emilia via G. Campi 287 Modena 41125 Italy

**Keywords:** bioresorbable chemical sensor, fluorescence sensor, implanted chemical sensor, in vivo pH sensor, layer‐by‐layer (LbL), porous silicon, wireless sensor

## Abstract

Here, the authors report on the manufacturing and in vivo assessment of a bioresorbable nanostructured pH sensor. The sensor consists of a micrometer‐thick porous silica membrane conformably coated layer‐by‐layer with a nanometer‐thick multilayer stack of two polyelectrolytes labeled with a pH‐insensitive fluorophore. The sensor fluorescence changes linearly with the pH value in the range 4 to 7.5 upon swelling/shrinking of the polymer multilayer and enables performing real‐time measurements of the pH level with high stability, reproducibility, and accuracy, over 100 h of continuous operation. In vivo studies carried out implanting the sensor in the subcutis on the back of mice confirm real‐time monitoring of the local pH level through skin. Full degradation of the pH sensor occurs in one week from implant in the animal model, and its biocompatibility after 2 months is confirmed by histological and fluorescence analyses. The proposed approach can be extended to the detection of other (bio)markers in vivo by engineering the functionality of one (at least) of the polyelectrolytes with suitable receptors, thus paving the way to implantable bioresorbable chemical sensors.

## Graphical abstract

In this work in vivo operation, biocompatibility, and full degradation of a nanostructured pH sensor are demonstrated. The sensor leverages a nanometer‐thick multilayer stack of polyelectrolytes labelled with a pH‐insensitive fluorophore conformably coated within a porous silica membrane—thickness of a few micrometers—to boost fluorescence up to 600 times and enable reliable measurements through skin.

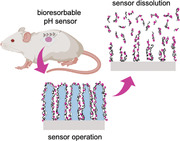

## 1 Introduction

In vivo chemical sensing has the potential to revolutionize healthcare providing access to the continuous monitoring of drugs and analytes trafficking in peripheral blood and tissues, and in turn to individualized reports on both disease progression and drug efficacy in real‐time.^[^ ^1^ ^]^ This would positively impact the way we diagnose diseases and monitor drug efficacy enabling personal, timely, and effective medical feedback, that is, a real‐time personalized medicine. The present‐day paradigm used in clinical practice based on single‐moment‐in‐time tests, that is, marker detection through ex‐situ analysis of extracted biofluids, has proven to be not always adequate or timely to respond to clinical needs.^[^ ^2^ ^]^

Bioresorbable materials^[^ ^2^, ^3^ ^]^ provide a unique opportunity to engineer new electrical, optical, and sensing components into an in vivo biodegradable sensing system that eliminates any boundary between target molecules and sensing devices, granting direct access to biofluids and acting as an in situ sentinel once implanted in the body, without the need of secondary device‐retrieval surgery that may cause tissue lesion or infection^[^ ^4^, ^5^ ^]^—often required for long‐lived implants, for example, pacemakers and cochlear implants. Chemical (bio)sensors made of bioresorbable materials have a huge potential in personalized medicine, especially for those treatments that benefit of a continuous and localized monitoring of specific analytes for a limited time, for example, chemotherapeutic cancer treatment, post‐surgery sepsis, acute trauma treatment, pharmacokinetics profiling, and disease biomarker detection.

Recently, in vivo transient electronic^[^ ^6^, ^7^, ^8^ ^]^ and photonic^[^ ^9^, ^10^, ^11^ ^]^ devices, as well as bioresorbable physical sensors^[^ ^12^, ^13^, ^14^, ^15^ ^]^ exploiting changes of the material properties (e.g., resistance, current) upon pressure or temperature variations have been reported to monitor intracranial temperature, pressure in orthopedic application, neural activity, tissue temperature, and cardiovascular signals. These devices are typically encapsulated with a suitable coating designed to guarantee—ideally—measurements in vivo for a prescribed time, then degrade hydrolytically over time following normal metabolism with negligible effects on local tissues. However, control over transiency is still at an early stage and relies on in vitro degradation studies of the encapsulation coating and device materials under specific conditions.^[^ ^16^, ^17^ ^]^ Further, chemical sensing with bioresorbable devices has shown to be challenging given that the necessary interaction of the sensing material with the target analyte forbids full encapsulation of the sensor.^[^ ^18^, ^19^ ^]^ Only a few examples of bioresorbable chemical sensors have been reported to date and demonstrated in vitro. Hwang et al.^[^ ^20^ ^]^ proposed a bioresorbable pH sensor using a field effect transistor‐like structure based on silicon nanoribbons. An organic, bioresorbable (yet not completely) electrochemical biosensor for the detection of glucose was reported by Pal et al.^[^ ^21^ ^]^ Kim et al.^[^ ^22^ ^]^ proposed a flexible, bioresorbable electrochemical sensor that employed silicon nanomembranes coated with iron‐containing nanoparticles as catalyst for the detection of neurotransmitters (i.e., dopamine). Bai et al.^[^ ^9^ ^]^ recently reported on flexible infrared waveguides made of thin filaments of Si nanomembranes with a poly(lactic‐co‐glycolic acid) (PLGA) cladding.

To the best of our knowledge, no demonstration of in vivo operation of bioresorbable chemical or pH sensors has been given to date.^[^ ^2^, ^23^ ^]^

Among the many analytes of clinical interest, the concentration of H^+^, that is, the pH value, is of utmost importance for acid‐base regulation.^[^ ^24^, ^25^, ^26^ ^]^ The concentration of H^+^ in blood plasma and various other body fluids is among the most tightly regulated variables in human physiology. Acute changes in blood pH induce powerful regulatory effects at the level of the cell, organ, and organism.^[^ ^27^ ^]^ For instance, the alteration of the pH value is predictive of cancer growth^[^ ^28^, ^29^ ^]^ and cardiac disease,^[^ ^30^ ^]^ among others. Tissue acidosis is a hallmark of inflammatory diseases. The acidification within diseased tissues is likely caused by cell death and hyperactive inflammatory cells. For instance, high H^+^ concentrations were found in acute wounds (i.e., pH of 5.4),^[^ ^31^ ^]^ in fracture‐related hematomas (i.e., pH of 4.7),^[^ ^32^ ^]^ and cancer microenvironment (i.e., pH of 6.4 to 7.3 in human melanoma).^[^ ^33^ ^]^

Recently, implantable pH sensors have attracted great attention thanks to the possibility of monitoring pH level in real time in humans.^[^ ^30^, ^34^ ^]^ Among these, optical pH sensors have been mainly proposed for point measurements in tissues and organs; pH‐responsive fluorescent probes, polymers, and nanoparticles have been used for intracellular pH mapping.^[^ ^34^ ^]^ Optical pH sensing is based on distinct absorption or fluorescence changes of appropriate pH indicators upon their protonation/deprotonation at different pH values. Compared to the absorption‐based pH indicators, fluorescence‐based pH indicators provide higher sensitivity and need a much lower concentration of the indicator. Many fluorescent small molecules have been successfully employed as pH indicators in non‐invasive and real‐time imaging of pH regulation in several physiological and pathological processes. However, fluorescent pH indicators show limited response ranges and many of them suffer from low solubility in aqueous solutions and significant toxicity.^[^ ^35^ ^]^ Regardless of the approach used, implantable pH sensors are typically affected by the lack of reliable performance in vivo, especially in terms of stability and reproducibility, due to both material degradation and body autoimmune response.^[^ ^30^, ^34^ ^]^

Herein, we report on the design, fabrication, and in vivo assessment of a bioresorbable nanostructured pH sensor. The sensor consists of a nanostructured porous silica scaffold conformably coated via layer‐by‐layer (LbL) assembling^[^ ^36^ ^]^ with a multilayer stack of two polyelectrolytes labelled with a pH‐insensitive fluorophore. The sensor fluorescence changes linearly with the pH value in the range 4 to 7.5 thanks to swelling/shrinking of the polymer stack and enables performing real‐time pH measurements with high reproducibility and resolution, over 100 h of continuous operation. Studies on an animal model are carried out implanting the sensor in the subcutis on the back of the animal and confirm in vivo real‐time measurement of the local pH value by monitoring fluorescence changes through skin. Full degradation of the pH sensor occurs in one week from implant in the animal model, and biocompatibility at 2 months is confirmed through histological and fluorescence analyses.

## 2 Results and Discussion

**Figure 1:**
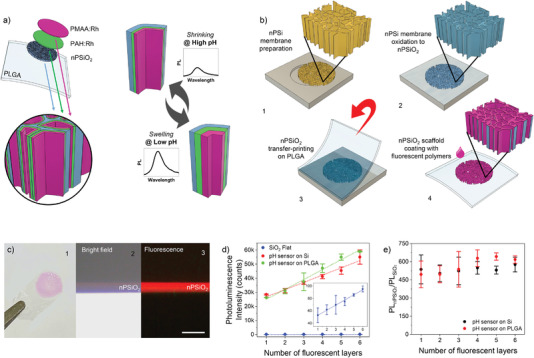
Preparation of the bioresorbable fluorescence pH sensor. a) Sketch of the pH sensor architecture and operation principle. The sensor consists of a micrometer‐thick nanostructured porous silicon oxide (nPSiO_2_) membrane coated with a nanometer‐thick pH‐responsive stack of two polymers labelled with Rh fluorophores (PAH:Rh and PMAA:Rh). b) Main fabrication steps of the pH sensor on PLGA substrate: 1) preparation of a ≈5‐µm‐thick nPSi membrane via two‐steps electrochemical silicon etching; 2) thermal oxidation of the nPSi membrane to nPSiO_2_; 3) transfer‐printing of nPSiO_2_ membrane onto a ≈40‐µm‐thick PLGA film; 4) layer‐by‐layer conformal coating of the nPSiO_2_ scaffold with a nanometer‐thick multilayer stack of PAH:Rh and PMAA:Rh. c) 1) Picture of the bioresorbable pH sensor highlighting the Rh‐coated nPSiO_2_ membrane (pink area) on the PLGA foil; 2) bright‐field and 3) fluorescence optical microscope images of the cross‐section of the nPSiO_2_ scaffold LbL‐coated with Rh‐labelled polyelectrolytes. Scale bar is 10 µm. d) Photoluminescence intensity at 580 nm versus number of Rh‐labelled polyelectrolyte layers assembled in the nPSiO_2_ scaffold either transfer‐printed on PLGA foil or left on the native Si chip, as well as on flat SiO_2_ substrate used as control. The inset highlights the photoluminescence intensity measured on flat SiO_2_ substrate (*n* = 3 samples for each polyelectrolyte architecture). e) Ratio of the photoluminescence intensity (at 580 nm) achieved on nPSiO_2_ scaffolds and flat PSiO_2_ substrate (i.e., PL_nSiO2_/PL_SiO2_) versus number of Rh‐labelled polyelectrolytes of the multilayer stack (*n* = 3 samples for each polyelectrolyte architecture). All data are presented as mean (± s.d).

The bioresorbable pH sensor consists of a micrometer‐thick membrane of nanostructured porous silica conformably coated with a nanometer‐thick pH‐responsive multilayer of two polymers labelled with Rhodamine‐B (Rh) fluorophore. The fluorescence intensity, upon excitation, reduces/increases linearly with the pH value of the environment surrounding the sensor, due to shrinking/swelling of the multilayer stack as the pH value changes. A sketch of the pH sensor architecture and of its operating principle are given in **Figure** **1**a.

**Figure 2:**
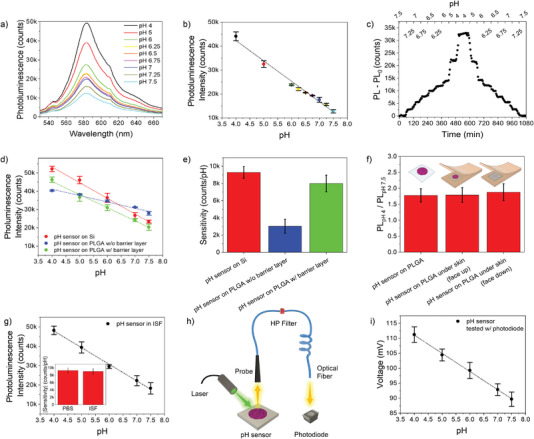
Assessment of the bioresorbable pH sensor in vitro. a) Photoluminescence spectra of the bioresorbable pH sensor measured in the pH range 4–7.5 in PBS at 37 °C. b) Calibration curve (photoluminescence intensity at 580 nm versus pH value) of the bioresorbable pH sensor measured over the pH range 4–7.5 in PBS at 37 °C (*n* = 3 pH cycles). c) Real‐time measurement of the photoluminescence intensity of the pH sensor at different pH values from 7.5 to 4, and back, in PBS at 37 °C. pH variation in the physiological range 6.5–7.5 is of 0.25 points. d) Calibration curve of the pH sensors in different configurations, namely, LbL‐coated nPSiO_2_ on native silicon chip (red) and on a PLGA film (blue), LbL‐coated nPSiO_2_ with barrier layer on a PLGA film (green) (*n* = 3 pH cycles). e) Sensitivity values of the sensors in (d) (*n* = 3 samples, 3 full cycles per sample). f) Photoluminescence intensity changes between pH 4 and 7.5 measured on the pH sensor in solution and between artificial skin flaps in two different configurations. (*n* = 3 samples per architecture, 3 full cycles per sample). Two‐tailed Student's t‐test (significance level < 0.01) confirmed that data were statistically equivalent. g). Calibration curve (photoluminescence intensity at 580 nm versus pH value) of the bioresorbable pH sensor measured over the pH range 4–7.5 in ISF at 37 °C (*n* = 3 pH cycles). Inset show the comparison of sensitivity values of the pH sensor in PBS and ISF (*n* = 3 samples, 3 full cycles per sample). h) Sketch of the setup used to perform pH sensing with a compact and cheap light source‐photodiode pair. i) Calibration curve (photodiode response versus pH value) of the bioresorbable pH sensor in the range of pH 4–7.5 measured with the setup in h) (*n* = 3 samples, 3 full cycles per sample). Data are presented as mean (± s.d).

**Figure 3:**
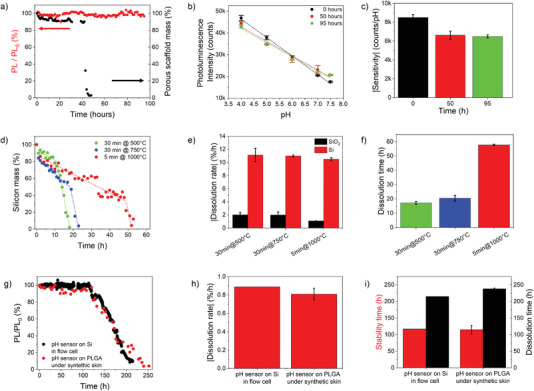
Long‐term operation of the bioresorbable pH sensor. a) Normalized photoluminescence intensity (red, at 580 nm) and mass of the porous scaffold (black) measured over ≈100 h on a pH sensor in buffer at pH 7.4 and 37 °C. b) Calibration curve (photoluminescence intensity at 580 nm versus pH value) of the pH sensor in (a) measured over the pH range 7.4–4 at *t* = 0, 50, and 95 h (*n* = 3 full cycles). c) Sensitivity value of the pH sensor in (b). d) Mass percentage of nPSiO_2_ scaffolds oxidized in different conditions measured over time until full dissolution. The raw data are best‐fitted using a piecewise linear model (dotted traces). e) Dissolution rate of the SiO_2_ capping layer and silicon skeleton of the nPSiO_2_ scaffolds in (d) (*n* = 4 samples). f) Full dissolution time of the nPSiO_2_ scaffolds in (d) (*n* = 3 samples). g) Time‐resolved normalized photoluminescence intensity (at 580 nm) of pH sensors on native Si in a flow cell and on PLGA foil under synthetic skin. h) Degradation rate (after 110 h of stable operation) of the photoluminescence of pH sensors on native Si in flow cell and on PLGA foil under synthetic skin (*n* = 3 samples). i) Photoluminescence stability and degradation times of pH sensors on native Si in flow cell and on PLGA foil under synthetic skin (*n* = 3 samples). Data are presented as mean (± s.d).

**Figure 4:**
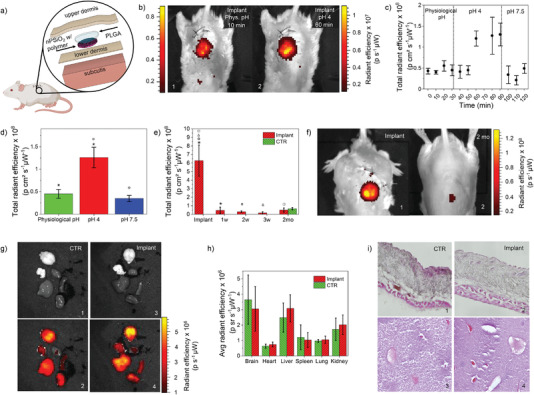
Assessment of pH sensing in vivo, bioresorbability, and biocompatibility. a) Sketch of the pH sensor implant in the animal model. b) In vivo fluorescent images acquired through skin on one of the animals implanted with the sensor on their back (excitation 520–560 nm, collection 620 nm), collected in 1) physiological conditions and 2) after local injection of a PBS solution at pH 4. c) In vivo real‐time fluorescence intensity signal measured through skin on mice implanted with the sensor, acquired in physiological conditions and after injection of PBS at pH 4 and 7.5 around the implant site (*n* = 3 mice). d) In vivo steady‐state fluorescence intensity measured through skin on mice implanted with the sensor, acquired in physiological conditions and after injection of PBS at pH 4 and 7.5 around the implant site (*n* = 3 mice). Symbols indicate statistically independent values (two‐tailed Student's *t*‐test, significance level <0.01). e) In vivo fluorescence intensity measured through skin on mice implanted with the sensor (*n* = 3 mice) and control mice (*n* = 5 mice) at different time points, before sacrifice. Symbols indicate statistically independent values (two‐tailed Student's *t*‐test, significance level <0.01). f) In vivo fluorescence images acquired through skin right after implant and after 2 months from implant. g) In vivo grayscale and fluorescent images of explanted organs (brain, heart, liver, spleen, lung, and kidney) of control mice (*n* = 5 mice) and mice implanted with the sensor (*n* = 6 mice), acquired after 2 months from implant. h) Mean fluorescence intensity of each organ in g) for the two groups of mice (control and implanted with sensor). i) Optical microscope images of skin (1,2) and liver (3,4) cryosections labeled with eosin and hematoxylin staining of a control mouse and a mouse implanted with the sensor after sacrifice at 2 months from implant. Data are presented as mean (± s.d).

The bioresorbable pH sensor is fabricated as sketched in Figure 1b and detailed in the Experimental Section. We prepared a free‐standing ≈5‐µm‐thick nanostructured porous silicon (nPSi) membrane with pores of ≈35 nm in size via a two‐steps electrochemical etching of a silicon wafer^[^ ^37^, ^38^ ^]^ (Figure 1b‐1 and Figure S1a–c, Supporting Information). The nPSi membrane was thermally oxidized at 1000 °C (nPSiO_2_) to enhance hydrophilicity and stability of the nanostructure in physiological conditions^[^ ^39^ ^]^ (Figure 1b‐2 and Figure S1d, Supporting Information). The nPSiO_2_ membrane was then transfer‐printed onto ≈40‐µm‐thick PLGA foil (Figure 1b‐3 and Figure S1e, Supporting Information). Eventually, we conformably coated the inner pore surface of the nPSiO_2_ membrane with a nanometer‐thick pH‐responsive polymer stack via layer‐by‐layer deposition.^[^ ^40^, ^41^, ^42^ ^]^ The polymer stack was assembled leveraging the negative surface charge of the nPSiO_2_ scaffold for the electrostatic deposition of alternating positive/negative polyelectrolytes labelled with Rh, namely, poly‐allylamine‐hydrochloride (PAH:Rh) and poly‐methacrylic‐acid (PMAA:Rh) (Figure 1b‐4). PAH and PMAA were selected among other weak polyelectrolytes due to their extensive use in vivo and well‐known biocompatibility.^[^ ^43^, ^44^ ^]^ Figure 1c shows the bioresorbable pH sensor on a PLGA foil. Optical microscope images in bright‐field and fluorescence mode of the sensor cross‐section reveal that the nPSiO_2_ membrane is uniformly coated with the Rh‐labelled polyelectrolytes. The photoluminescence (PL) emission of the LbL polymer stack is peaked at ≈580 nm, in agreement with Rh emission^[^ ^45^ ^]^ (Figure S2, Supporting Information).

The incorporation of fluorophores into nano‐sized biocompatible matrices, such as organic and inorganic nanoparticles, nanostructures, and quantum dots, is known to reduce toxicity and solubility of the fluorophores, improve their photostability, and extend the response range.^[^ ^35^ ^]^ We leveraged the augmented surface‐to‐volume ratio (550× compared to a flat substrate) of the nanostructured porous scaffold and the conformal coating of its high aspect‐ratio pores (length‐to‐width ≈100) to increase the number of Rh molecules per unit area and, in turn, the intensity of the fluorescence emission, with respect to a flat substrate. The fluorescence intensity increased linearly with the nPSi thickness and in turn surface, once a constant porosity is set (Figure S3, Supporting Information). We found that the fluorescence intensity of the Rh‐coated nPSiO_2_ membrane on PLGA foil was ≈600 times higher than that of the flat Rh‐coated SiO_2_ substrate, regardless of the number of fluorescent layers in the polymer stack (Figure 1d,e). Thus, we have flexibility in tuning the number of layers in the stack to optimize the sensing performance of the latter. Control samples prepared by coating nPSiO_2_ scaffold with the fluorescent polymer stack directly on the native silicon chip (i.e., without peeling the membrane off the silicon substrate) (Figure S4, Supporting Information) showed no differences in terms of fluorescence amplification with respect to nPSiO_2_ scaffolds transfer‐printed on PLGA foil (Figure 1d,e). This is an indication that both transfer‐printing and polymer coating processes of the nPSiO_2_ membrane on PLGA foils are robust and reliable.

We carried out a set of experiments on nPSiO_2_ scaffolds on Si chips in different assembly conditions and number of layers of the polymer stack to optimize sensitivity to pH. The use of TRIS at pH 8 as the assembly buffer ensured full ionization of both PAH^[^ ^46^ ^]^ and PMAA^[^ ^47^ ^]^ and, in turn, the highest sensitivity to pH, once the number of layers was chosen (Figure S5, Supporting Information). TRIS at pH 8 was used as the assembly buffer from then on, if not differently stated. The number of PAH:Rh and PMAA:Rh layers in the stack was then varied from (PAH:Rh/PMAA:Rh)_1_ to (PAH:Rh/PMAA:Rh)_3_+(PAH:Rh)_1_. The thickness of the polymer stack increased linearly with the number of layers reaching a maximum value of ≈8 nm, thus leaving the pores sufficiently open to ensure effective H^+^ diffusion within the porous scaffold (Figure S6a, Supporting Information). Conformal assembling of the polymer stack in the porous scaffold is supported by the cumulative red‐shift of the nPSiO_2_ effective optical thickness (EOT) with the number of layers (Figure S6b,c, Supporting Information).

For all the polymer stack architectures the sensor response was investigated in the pH range 4 to 7.5 (3 samples, 3 full cycles per each sample) at physiological temperature (37 °C) in PBS. A linear relationship between photoluminescence intensity and pH value is achieved regardless of the stack architecture. Reproducibility of the sensor performance both in‐sample and from sample‐to‐sample (over repeated cycles) is excellent, with a pH resolution < 0.1 points in the physiological range 6–7.5 (**Figure** **2**a,b). The fluorescence intensity reliably decreases as the pH value increases and vice versa. Variation of the polyelectrolyte ionization degree and in turn of the cohesion of the polyelectrolytes in the stack with pH changes leads to a shrinking/swelling of the polymers that reduces/increases the distance of nearest neighbor Rh fluorophores (Figure 1a). Consequently, Rh photoluminescence self‐quenching reduces as the pH increases and vice versa.

Shrinking/swelling of the polymer stack as the sensing mechanism was confirmed by investigating photoluminescence changes of PAH:Rh and PMAA:Rh solutions at different pH values in the range 4–7 (Figure S7, Supporting Information). No significant photoluminescence changes with pH were apparent for the polyelectrolyte solutions.

Time‐resolved kinetics of the sensor was next investigated by real‐time monitoring of the photoluminescence intensity upon injection of a buffer with different pH values in the range 4–7.5. The sensor photoluminescence intensity quickly changes upon variation of the pH value of the buffer, reaching a new steady‐state value in ≈20 min (Figure 2c). We found that the architecture of the polymer stack did not affect the response time but impacted significantly on the sensitivity of the sensor to pH (Figure S8a–c, Supporting Information). The sensitivity is maximum when an odd number of polymers is assembled within the porous scaffold, that is, (PAH:Rh/PMAA:Rh)_x_+(PAH:Rh)_1_ with *x* ranging from 1 to 3. In this case the sensitivity value is constant regardless of the number of layers assembled in the stack (Figure S8a, Supporting Information). Conversely, when an even number of polymers is assembled in the stack, that is, (PAH:Rh/PMAA:Rh)_x_ with *x* ranging from 1 to 3, the sensitivity to pH increases linearly with the number of layers, yet remaining lower than that of the odd stack (Figure S8b, Supporting Information). This behavior is explained in terms of different electroneutrality requirements and different osmotic pressures inside the polymer stack with even and odd number of layers that regulates the swelling of the LbL nano‐coating.^[^ ^48^, ^49^ ^]^

Layer‐by‐layer functionalization of nPSiO_2_ membranes transfer‐printed on PLGA foils resulted in no substantial changes of the sensing performance, apart from a reduction of the sensitivity due to partial infiltration of the pores with PLGA that limits swelling of the polymer stack (Figure S9, Supporting Information). The sensitivity reduction was successfully recovered inhibiting the diffusion of PLGA inside the pore structure by preparation of a nPSiO_2_ membrane with an additional 100‐nm‐thick barrier layer at its bottom with low porosity (≈61%) and tiny pores (≈7 nm) in contact with the PLGA foil (Figure 2d,e).^[^ ^50^ ^]^

To mimic in vivo application, the pH sensor was placed between two synthetic skin flaps (Figure 2f inset) conditioned overnight in PBS at pH 7.4. Two different configurations of the sensor were considered, namely, with the pH‐responsive fluorescent polymer stack face‐up (i.e., directed toward the spectrometer probe) or face‐down. Whereas the face‐up operation guarantees an increased photoluminescence intensity collected through skin, the face down operation is more suitable for in vivo subdermal application as the sensing stack is in contact with blood vessels, lymphatic vessels, nerves, and glands that connect it to the dermis.^[^ ^51^ ^]^ Photoluminescence spectra acquired through the artificial skin highlight a factor 2 change in intensity between the two configurations; yet the line‐shape of the spectra is unchanged, with skin autofluorescence appreciable at ≈550 nm (Figure S10a,b, Supporting Information). The photoluminescence emission of the sensor is stable with time in physiological conditions. When the pH of the synthetic skin is locally changed by intradermal injection of a PBS solution at pH = 4, a steady increase of the PL intensity occurs (Figure S10c, Supporting Information). Once the PL intensity has reached the steady state value at pH = 4, injection of a PBS solution at pH = 7.5 reduces the PL emission back to its initial value. Figure S10d, Supporting Information, shows steady‐state values of the PL intensity for cyclic pH changes between 7.5 to 4, highlighting a good reproducibility of the pH sensor operation under synthetic skin. Despite the higher PL intensity of the face‐up configuration (Figure S10e, Supporting Information), same PL intensity variation, namely, a factor ≈1.8, was retrieved for both the configurations (Figure 2f). This value is statistically equivalent to the variation measured for the sensor immersed in liquid solutions.

We then performed pH sensing experiments using synthetic interstitial fluid (ISF)^[^ ^52^ ^]^ as a complex matrix to mimic in vivo operation. No significant differences were found between sensors tested in PBS and in ISF, despite the complexity of ISF that contains proteins, *α*‐globulins, and salts. A linear response between PL intensity and pH value was achieved also in ISF, with high sample‐to‐sample and in‐sample reliability (Figure 2g). The sensitivity values in ISF and in PBS were statistically equivalent (3 sensors, 3 full cycles per sensor) (Figure 2g inset). Interestingly, the ratio of the PL intensity measured at pH 4 and 7.5 in ISF was 2.83 ± 0.28. The larger ratio in ISF compared to PBS can be explained with the infiltration of the polymer stack with proteins, in agreement with the literature^[^ ^53^ ^]^ (Figure S11, Supporting Information).

A set of experiments was further performed to demonstrate that the pH sensor can be operated using a cheap and compact system exploiting a light source‐photodiode pair, besides the use of more expensive and cumbersome conventional spectrometers and/or imaging systems (Figure 2h and Figure S12, Supporting Information). Specifically, we replaced the spectrometer with a photodiode (5 mm × 3 mm × 2.1 mm) with peak sensitivity at 560 nm to collect the PL intensity of the sensor at different pH levels and used an in‐line long‐pass filter with a cut‐off wavelength of 515 nm coupled to the fiber‐optic probe to remove the excitation light of the laser (1.1 cm × 4 cm). The system allowed measuring pH over the range 4–7.5 with excellent linearity, high sensitivity (−6.2 ± 1 mv/pH), and excellent sample‐to‐sample and in‐sample reliability (CV% = 2.3%), as shown in the Figure 2i.

Long‐term operation was investigated by real‐time measurement of the pH sensor photoluminescence over more than 200 h of continuous operation, using a flow cell injected with buffer at different pH values in the range 7.5 to 4 and constant temperature of 37 °C. The pH of the buffer solution was maintained constant at 7.4 over the full time span, apart from specific time intervals starting at *t* = 0, 50, and 95 h during which the pH of the solution was varied in the range 7.5–4, and back (3 full cycles) with steps of 0.5 points. The sensor photoluminescence is stable over ≈100 h of operation, namely, 114 h (±std 13 h), with a normalized intensity PL/PL_0_ = 96.6% (±std 2.2%), being PL_0_ the intensity at *t* = 0 h (**Figure** **3**a). Calibration curves of the sensor recorded at *t* = 0, 50, and 95 h from the beginning of the experiment are given in Figure 3b. The sensor response to pH is reliable over time (CV% = 4.3%), keeping high linearity and good sensitivity after 100 h of operation (Figure 3b,c).

Beside photoluminescence, the reflectance spectrum of the pH sensor was simultaneously measured to retrieve information on the real‐time dissolution of the porous scaffold (Figure 3a). Fast Fourier Transformation (FFT) of the reflectance spectrum of a bare nPSiO_2_ scaffold results in a signal whose intensity is proportional to the mass of the porous scaffold (Figure S13, Supporting Information). Given that fluorescence emission of the sensor is stable for a time of 100 h, at least, one can assume that the polymer stack is also stable. Thus, changes occurring in the intensity of the FFT signal with time can be directly linked to the dissolution of the porous scaffold in the pH sensor.^[^ ^54^ ^]^ We found that dissolution of the nPSiO_2_ scaffold occurs in ≈50 h (Figure 3a). Remarkably, fluorescence stability (Figure 3a) and sensing properties (Figure 3b) of the polymer stack are not affected by the scaffold dissolution. The pH sensor reduces to a 6‐nanometer‐thick polymer brush with 5‐µm‐long wire bristles after dissolution of the silica scaffold occurring in ≈2 days, further lessening invasiveness and improving adaptability of the sensor to tissue, yet retaining its mechanical integrity and in turn PL and sensing properties for at least 2 more days (Figure S14, Supporting Information).

The dissolution time achieved for the porous scaffold in the pH sensor (Figure 3a) was consistent with that of a bare nPSiO_2_ scaffold oxidized at 1000 °C for 5 min (Figure 3d). No statistical differences were observed between dissolution times of polymer‐coated and bare scaffolds (Figure S15, Supporting Information). Control experiments on dissolution were carried out on bare nPSiO_2_ scaffolds oxidized in different conditions, in buffer at pH 7.4 and 37 °C (Figure S16a–c, Supporting Information). A progressive reduction of both fringe contrast and the effective optical thickness (EOT) values of the reflectance spectrum was observed with time (Figure S16d–f, Supporting Information). The FFT peak intensity retrieved from the reflectance spectrum of the bare nPSiO_2_ scaffold (i.e., without any polymer coating) was used to estimate the silicon content in the scaffold over time. The dissolution kinetics of nPSiO_2_ was best‐fitted with a piece‐wise linear model with two slopes, from which it was possible to extrapolate the dissolution rates of the SiO_2_ capping layer (first slope) and of the residual Si skeleton in the scaffold (second slope) (Figure 3d). The dissolution rate of SiO_2_ reduced (from ≈2%/h to 1%/h at 500 and 1000 °C, respectively) as the oxidation temperature increased, consistently with the higher quality of the oxide layer; a faster dissolution rate of ≈10.5%/h was achieved for the silicon skeleton (Figure 3e). The transition time between the two dissolution regimes also changed with the oxidation temperature from ≈10 h at 500 °C to 52 h at 1000 °C, due to the slower dissolution rate and larger thickness of SiO_2_ (Figure S17, Supporting Information).

After ≈114 h of continuous operation at pH 7.5 the photoluminescence intensity of the sensor starts decreasing with a linear trend, then vanishes (signal‐to‐noise ratio of 3.3) after ≈220 h (Figure 3g–i). The PL intensity decrease can be ascribed to the degradation, chemical and/or mechanical, of the fluorescent polymer multilayer, given that the silica scaffold dissolved in ≈50 h. The polymer brush architecture of the fluorescent multilayer after dissolution of the silica scaffold might impact significantly on its degradation (Figure S14, Supporting Information). Remarkably, full sensor degradation occurred on the same timescale as the sensor operation, that is, in ≈100 h. By best fitting the PL reduction trend with a linear model, degradation rates of about −0.9%/h and −0.8%/h were consistently achieved for pH sensors on native silicon in a flow cell and on PLGA foil under artificial skin, respectively. The polymer multilayer degradation was further confirmed by both optical/fluorescence microscopy. No macroscopic polymer residues or residual photoluminescence were found in the solution collected at the output of the flow cell or in the skin around the sensor location at the end of the experiment, indicating that a full degradation of the polymer and in turn of the sensor occurred, apart from the PLGA foil that is known to degrade at a slower rate.^[^ ^2^ ^]^

We next carried out in vivo studies in an animal model to assess sensing performance and biocompatibility/bioresorbability. The pH sensor was implanted in the subcutis on the back of a group of adult male and female mice, as sketched in **Figure** **4**a. Another group was not implanted with the sensor and used as control. Experimental procedures on animals were done in accordance with the European Community regulations on the use and care of animals for scientific purposes.^[^ ^44^ ^]^

A subgroup of the animals implanted with the sensor was used to perform real‐time pH measurements through skin. Fluorescence images were acquired on this group of mice every 10 min, starting 15 min after the implant, over a time span of 2 h using an in vivo animal imager system. Figure 4b shows typical fluorescence images acquired on one of the animals implanted with the pH sensor in physiological conditions and at pH 4. The implant location and fluorescence emission of the sensor are clearly visible, with the fluorescence intensity increasing as pH reduces. The sensor fluorescence was stable during the first 30 min of acquisition in physiological conditions, despite possible coagulation and immune response processes occurring at the implant site right after surgery (Figure 4c). A PBS solution with pH = 4 was injected a *t* = 30 and 60 min in the subcutaneous tissue around the implant site, with the aim of lowering the pH value in the subcutis. After a lag time of 30 min from the first injection at pH 4 we observed a neat increase of the sensor fluorescence, with an average incremental factor of ≈2.8 compared to physiological conditions (Figure 4c,d). Remarkably, this incremental factor is in perfect agreement with that measured in vitro in ISF, namely, 2.83 ± 0.28 (Figure S18, Supporting Information). A further injection of the pH 4 solution at *t* = 60 min did not produce any significant change in the fluorescence level acquired after the first injection, indicating that the pH level of the tissue around the sensor implant was stable at 4. The fluorescence emission was stable until a PBS solution with pH = 7.5 was further injected at *t* = 90 min in the subcutis around the sensor to induce the rebalancing of the local pH of the mouse. After 10 min from the injection, the fluorescence emission returned to the initial value measured in physiological conditions. The faster recovery time is consistent with the faster dose‐response time of alkaline solutions in comparison with acidic solutions.^[^ ^45^ ^]^

A second subgroup of the mice implanted with the sensor was used to investigate sensor degradation with time. Fluorescence images were acquired on the animals right after implantation of the sensor, then after 7, 14, 21, and 60 days. The sensor fluorescence dropped after 1 week from implant to a level that was statistically equal to the tissue autofluorescence measured on the control group (Figure 4e). The fluorescence images acquired on the animals consistently pointed out a reduction of both emission area and intensity at the implant site that saturated to the tissue autofluorescence (Figure 4f). We speculate that these data are consistent with a full degradation and/or bioresorption of the sensor.

The two groups of animals (both with and without sensor implanted) were sacrificed after 2 months from implant to verify biocompatibility of the pH sensor following the UNI EN ISO10993‐6 guidelines.^[^ ^55^ ^]^ We performed a macroscopic evaluation of the surgical area using a 4‐point scale (0 = intact skin; 1 = swelling, 2 = redness, 3 = eschar) and a microscopic evaluation by sampling of a square skin area adjacent to the implant (1.5 cm × 1.5 cm) and organs (liver, kidney, heart, brain, lung, and spleen) for histological investigations.

Visual examination and fluorescence microscopy of the tissue at the implant site and around it did not show any residual of the sensor, thus corroborating the hypothesis of complete degradation of the sensor in 2 months, consistently with data in Figure 4e,f.

Fluorescence images of the organs were acquired right after extraction to investigate the presence of polymer residues in the group of animals implanted with the sensor. Figure 4g shows bright‐field and fluorescence images of brain, heart, liver, spleen, lung, and kidney of a control mouse and a mouse implanted with the pH sensor. No statistical difference in fluorescence emission was observed in any of the organs of the two groups, thus indicating that there is no significant polymer accumulated in the organs of mice implanted with sensors (Figure 4h).

The mass of Rh in our pH sensor is ≈1.2 µg, which corresponds to a dose of 0.024 mg/kg_BW_ for mice of 50 g used in this work. The Rh dose is at least 1000 times smaller than those tested for toxicity in vivo, namely, 22.5–100 mg/kg_BW_, and known to lead to oxidative stress, cell damage, and increase apoptosis in cerebellum tissue and brain stem.^[^ ^56^ ^]^ Figure 4i shows histological analysis images of the skin and liver morphology of a control mouse and a mouse implanted with the sensor. There are no morphological differences between the two groups, no cells show pycnosis, nuclear dust, swollen pericaryon, cell shrinkage, and the cell number is similar between the two groups (*n* = 6). The data prove that the sensor did not induce permanent injury and the tissue in contact with the sensor did not leave lesions or fibrotic areas. Similar consideration can be done also for liver. Macroscopic analysis also did not show differences between the two groups of animals (Figure S19, Supporting Information), just as no difference was observed in the weight of mice before surgical implant of the sensor and after 60 days (Figure S20, Supporting Information); this indicates that mice did not undergo significant physiological alterations, if there had been, during the 2 months of observation, the first consequence would have been a slowdown in the growth of the animal weight.

## 3 Conclusions

In this work we assessed the fabrication, in vivo operation, and biocompatibility of a bioresorbable nanostructured pH sensor that leverages a nanostructured porous silica membrane conformably coated layer‐by‐layer with a pH‐responsive stack of polyelectrolytes labelled with Rhodamine‐B. The calibration curve (fluorescence intensity versus pH value) is linear in the range 4 to 7.5. The sensor permits continuous real‐time measurements of the pH with good reproducibility (CV% = 4.3%) and resolution (<0.1 points) in vitro, over 100 h of continuous operation. In vivo studies carried out implanting the sensor in the subcutis on the back of mice assess the possibility of doing real‐time measurement of the local pH value monitoring fluorescence changes through skin. Full degradation of the pH sensor occurs in one week from implant, and biocompatibility is successfully assessed on animals implanted with the sensor for 2 months.

The proposed approach paves the way toward the development of bioresorbable chemical sensors for different analytes of clinical interest, besides pH, leveraging the fluorescence amplification of nPSiO_2_ membranes coupled with polyelectrolytes labelled with specific (bio)receptors and/or with different fluorophores to implement a multiwavelength sensing, for example, two‐wavelength ratiometric, fluorescence resonance energy transfer (FRET). The use of polyelectrolytes and porous scaffolds with higher stability and/or lower degradation rate in vivo, with respect those used in this work, can be leveraged to tailor the lifetime of the sensor to specific diseases by extending it beyond the 100 h.

## Conflict of Interest

The authors declare no conflict of interest.

## Supporting information

Supporting Information

## Data Availability

The data that support the findings of this study are available from the corresponding author upon reasonable request.

## References

[1] R. J. Soto , J. R. Hall , M. D. Brown , J. B. Taylor , M. H. Schoenfisch , Anal. Chem. 2017, 89, 276. 28105839 10.1021/acs.analchem.6b04251 PMC6773264

[2] A. A. La Mattina , S. Mariani , G. Barillaro , Adv. Sci. 2020, 7, 1902872. 10.1002/advs.201902872 PMC7029671 32099766

[3] G. D. Cha , D. Kang , J. Lee , D.‐H. Kim , Adv. Healthcare Mater. 2019, 8, 1801660.

[4] K. Bazaka , M. V. Jacob , Electronics 2012, 2, 1.

[5] H. C. Koydemir , A. Ozcan , Annu. Rev. Anal. Chem. 2018, 11, 127. 10.1146/annurev-anchem-061417-125956 29490190

[6] S.‐W. Hwang , H. Tao , D.‐H. Kim , H. Cheng , J.‐K. Song , E. Rill , M. A. Brenckle , B. Panilaitis , S. M. Won , Y.‐S. Kim , Y. M. Song , K. J. Yu , A. Ameen , R. Li , Y. Su , M. Yang , D. L. Kaplan , M. R. Zakin , M. J. Slepian , Y. Huang , F. G. Omenetto , J. A. Rogers , Science 2013, 337, 1640. 10.1126/science.1226325 PMC3786576 23019646

[7] S.‐W. Hwang , S.‐K. Kang , X. Huang , M. A. Brenckle , F. G. Omenetto , J. A. Rogers , Adv. Mater. 2015, 27, 47. 25357247 10.1002/adma.201403051

[8] J. Koo , M. R. Macewan , S.‐K. Kang , S. M. Won , M. Stephen , P. Gamble , Z. Xie , Y. Yan , Y.‐Y. Chen , J. Shin , N. Birenbaum , S. Chung , S. B. Kim , J. Khalifeh , D. V. Harburg , K. Bean , M. Paskett , J. Kim , Z. S. Zohny , S. M. Lee , R. Zhang , K. Luo , B. Ji , A. Banks , H. M. Lee , Y. Huang , W. Z. Ray , J. A. Rogers , Nat. Med. 2018, 24, 1830. 30297910 10.1038/s41591-018-0196-2

[9] W. Bai , H. Yang , Y. Ma , H. Chen , J. Shin , Y. Liu , Q. Yang , I. Kandela , Z. Liu , S.‐K. Kang , C. Wei , C. R. Haney , A. Brikha , X. Ge , X. Feng , P. V. Braun , Y. Huang , W. Zhou , J. A. Rogers , Adv. Mater. 2018, 30, 1801584. 10.1002/adma.201801584 PMC6148372 29944186

[10] D. Lu , T.‐L. Liu , J.‐K. Chang , D. Peng , Y. Zhang , J. Shin , T. Hang , W. Bai , Q. Yang , J. A. Rogers , Adv. Mater. 2019, 31, 1902739. 10.1002/adma.201902739 31489737

[11] R. Fu , W. Luo , R. Nazempour , D. Tan , H. Ding , K. Zhang , L. Yin , J. Guan , X. Sheng , Adv. Opt. Mater. 2018, 6, 1700941.

[12] G. A. Salvatore , J. Sülzle , F. Dalla Valle , G. Cantarella , F. Robotti , P. Jokic , S. Knobelspies , A. Daus , L. Büthe , L. Petti , N. Kirchgessner , R. Hopf , M. Magno , G. Tröster , Adv. Funct. Mater. 2017, 27, 1702390.

[13] X. Jia , C. Wang , V. Ranganathan , B. Napier , C. Yu , Y. Chao , M. Forsyth , F. G. Omenetto , D. R. Macfarlane , G. G. Wallace , ACS Energy Lett. 2017, 2, 831.

[14] J. Shin , Z. Liu , W. Bai , Y. Liu , Y. Yan , Y. Xue , I. Kandela , M. Pezhouh , M. R. MacEwan , Y. Huang , W. Z. Ray , W. Zhou , J. A. Rogers , Sci. Adv. 2019, 5, eaaw1899. 31281889 10.1126/sciadv.aaw1899 PMC6611687

[15] C. M. Boutry , A. Nguyen , Q. O. Lawal , A. Chortos , S. Rondeau‐Gagné , Z. Bao , Adv. Mater. 2015, 27, 6954. 26418964 10.1002/adma.201502535

[16] S.‐W. Hwang , G. Park , C. Edwards , E. A. Corbin , S.‐K. Kang , H. Cheng , J.‐K. Song , J.‐H. Kim , S. Yu , J. Ng , J. E. Lee , J. Kim , C. Yee , B. Bhaduri , Y. Su , F. G. Omennetto , Y. Huang , R. Bashir , L. Goddard , G. Popescu , K.‐M. Lee , J. A. Rogers , ACS Nano 2014, 8, 5843. 24684516 10.1021/nn500847g

[17] L. Yin , H. Cheng , S. Mao , R. Haasch , Y. Liu , X. Xie , S.‐W. Hwang , H. Jain , S.‐K. Kang , Y. Su , R. Li , Y. Huang , J. A. Rogers , Adv. Funct. Mater. 2014, 24, 645.

[18] J. Zhao , H. Guo , J. Li , A. J. Bandodkar , J. A. Rogers , Trends Chem 2019, 1, 559.

[19] Z. Morsada , M. M. Hossain , M. T. Islam , M. A. Mobin , S. Saha , Appl. Mater. Today 2021, 25, 101257.

[20] S.‐W. Hwang , C. H. Lee , H. Cheng , J.‐W. Jeong , S.‐K. Kang , J.‐H. Kim , J. Shin , J. Yang , Z. Liu , G. A. Ameer , Y. Huang , J. A. Rogers , Nano Lett. 2015, 15, 2801. 25706246 10.1021/nl503997m

[21] R. K. Pal , A. A. Farghaly , C. Wang , M. M. Collinson , S. C. Kundu , V. K. Yadavalli , Biosens. Bioelectron. 2016, 81, 294. 26985581 10.1016/j.bios.2016.03.010

[22] H. S. Kim , S. M. Yang , T. M. Jang , N. Oh , H. S. Kim , S. W. Hwang , Adv. Healthcare Mater. 2018, 7, 1801071. 10.1002/adhm.201801071 30450726

[23] Z. Wei , Z. Xue , Q. Guo , Micromachines 2021, 12, 600. 34067419 10.3390/mi12060600 PMC8224698

[24] G. N. Nakhoul , J. D. Schold , S. Arrigain , S. C. Harb , S. Jolly , B. L. Wilkoff , J. V. Nally , S. D. Navaneethan , Clin. J. Am. Soc. Nephrol. 2015, 10, 1119. 26111859 10.2215/CJN.11121114 PMC4491299

[25] V. L. Hood , R. L. Tannen , N. Engl. J. Med. 1998, 339, 819. 9738091 10.1056/NEJM199809173391207

[26] G. J. Casimir , N. Lefèvre , F. Corazza , J. Duchateau , M. Chamekh , Front Immunol 2018, 9, 475. 29593728 10.3389/fimmu.2018.00475 PMC5854649

[27] J. A. Kellum , M. Song , J. Li , Crit. Care 2004, 8, 331. 15469594 10.1186/cc2900 PMC1065014

[28] H. Izumi , T. Torigoe , H. Ishiguchi , H. Uramoto , Y. Yoshida , M. Tanabe , T. Ise , T. Murakami , T. Yoshida , M. Nomoto , K. Kohno , Cancer Treat. Rev. 2003, 29, 541. 14585264 10.1016/s0305-7372(03)00106-3

[29] S. Grinstein , D. Rotin , M. J. Mason , BBA – Rev Biomembr. 1989, 988, 73. 10.1016/0304-4157(89)90004-x 2535787

[30] M. T. Ghoneim , A. Nguyen , N. Dereje , J. Huang , G. C. Moore , P. J. Murzynowski , C. Dagdeviren , Chem. Rev. 2019, 119, 5248. 30901212 10.1021/acs.chemrev.8b00655

[31] Y.‐T. Tsai , J. Zhou , H. Weng , J. Shen , L. Tang , W.‐J. Hu , Adv. Healthcare Mater. 2014, 3, 221. 10.1002/adhm.201200365 23828849

[32] K. Steen , A. Steen , P. Reeh , J. Neurosci. 1995, 15, 3982. 7751959 10.1523/JNEUROSCI.15-05-03982.1995 PMC6578188

[33] Z. Boussadia , J. Lamberti , F. Mattei , E. Pizzi , R. Puglisi , C. Zanetti , L. Pasquini , F. Fratini , L. Fantozzi , F. Felicetti , K. Fecchi , C. Raggi , M. Sanchez , S. D'atri , A. Carè , M. Sargiacomo , I. Parolini , J Exp Clin Cancer Res 2018, 37, 245. 30290833 10.1186/s13046-018-0915-z PMC6173926

[34] A. Steinegger , O. S. Wolfbeis , S. M. Borisov , Chem. Rev. 2020, 120, 12357. 33147405 10.1021/acs.chemrev.0c00451 PMC7705895

[35] K.‐K. Yu , K. Li , J.‐T Hou , J. Yang , Y.‐M. Xie , X.‐Q. Yu , Polym. Chem. 2014, 5, 5804.

[36] J. J. Richardson , J. Cui , M. Björnmalm , J. A. Braunger , H. Ejima , F. Caruso , Chem. Rev. 2016, 116, 14828. 27960272 10.1021/acs.chemrev.6b00627

[37] S. Mariani , L. Pino , L. M. Strambini , L. Tedeschi , G. Barillaro , ACS Sens. 2016, 1, 1471.

[38] Y. Wan , N. A. Krueger , C. R. Ocier , P. Su , P. V. Braun , B. T. Cunningham , Adv. Opt. Mater. 2017, 5, 1700605.

[39] K.‐P. S. Dancil , D. P. Greiner , M. J. Sailor , J. Am. Chem. Soc. 1999, 121, 7925.

[40] S. Mariani , A. Paghi , A. A. La Mattina , A. Debrassi , L. Dähne , G. Barillaro , ACS Appl. Mater. Interfaces 2019, 11, 43731. 31644268 10.1021/acsami.9b15737

[41] S. Mariani , V. Robbiano , L. M. Strambini , A. Debrassi , G. Egri , L. Dähne , G. Barillaro , Nat. Commun. 2018, 9, 5256. 30531860 10.1038/s41467-018-07723-8 PMC6288083

[42] G. Decher , M. Eckle , J. Schmitt , B. Struth , Curr. Opin. Colloid Interface Sci. 1998, 3, 32.

[43] W. Gao , J. M. Chan , O. C. Farokhzad , Mol Pharm 2010, 7, 1913. 20836539 10.1021/mp100253e PMC3379544

[44] H. Gao , O. A. Goriacheva , N. V. Tarakina , G. B. Sukhorukov , ACS Appl. Mater. Interfaces 2016, 8, 9651. 27008032 10.1021/acsami.6b01921

[45] G. Ibarz , L. Dähne , E. Donath , H. Möhwald , Adv. Mater. 2001, 13, 1324.

[46] S. E. Burke , C. J. Barrett , Langmuir 2003, 19, 3297.

[47] L. Ruiz‐Pérez , A. Pryke , M. Sommer , G. Battaglia , I. Soutar , L. Swanson , M. Geoghegan , Macromolecules 2008, 41, 2203.

[48] B. Schwarz , M. Schönhoff , Langmuir 2002, 18, 2964.

[49] C. S. Peyratout , L. Dähne , Angew Chemie ‐ Int Ed. 2004, 43, 3762. 10.1002/anie.200300568 15258935

[50] S. Mariani , V. Robbiano , R. Iglio , A. A. La Mattina , P. Nadimi , J. Wang , B. Kim , T. Kumeria , M. J. Sailor , G. Barillaro , Adv. Funct. Mater. 2020, 30, 1906836. 32377177 10.1002/adfm.201906836 PMC7202556

[51] S. P. Commins , J. Allergy Clin. Immunol. 2015, 123, AB221.

[52] L. M. Strambini , A. Longo , S. Scarano , T. Prescimone , I. Palchetti , M. Minunni , D. Giannessi , G. Barillaro , Biosens. Bioelectron. 2015, 66, 162. 25601169 10.1016/j.bios.2014.11.010

[53] E. Bittrich , K. B. Rodenhausen , K.‐J. Eichhorn , T. Hofmann , M. Schubert , M. Stamm , P. Uhlmann , Biointerphases 2010, 5, 159. 21219037 10.1116/1.3530841

[54] S. P. Low , N. H. Voelcker , L. T. Canham , K. A. Williams , Biomaterials 2009, 30, 2873. 19251317 10.1016/j.biomaterials.2009.02.008

[55] UNI EN ISO10993‐6, http://store.uni.com/catalogo/uni‐en‐iso‐10993‐6‐2017?___store=en&josso_back_to=http%3A%2F%2Fstore.uni.com%2Fjosso‐security‐check.php&josso_cmd=login_optional&josso_partnerapp_host=store.uni.com.&___from_store=it. (accessed March 2022).

[56] C. Mahdi , C. A. Pratama , H. Pratiwi , IOP Conf Ser Mater Sci Eng 2019, 546, 062015.

