# Supplementary material for: Bioresorbable Nanostructured Chemical Sensor for Monitoring of pH Level In Vivo
Source: Adv Sci (Weinh). 2022 May 26;9(22):2202062. doi: 10.1002/advs.202202062 (PMC9353472; doi:10.1002/advs.202202062)
Supplement: Supplementary file 1 — Supporting Information [file ADVS-9-2202062-s001.pdf]

ADVANCED SCIENCE
Open Access

Supporting Information

for *Adv. Sci.*, DOI 10.1002/advs.202202062

Bioresorbable Nanostructured Chemical Sensor for Monitoring of pH Level In Vivo

*Martina Corsi, Alessandro Paghi, Stefano Mariani, Giulia Golinelli, Aline Debrassi, Gabriella Egri, Giuseppina Leo, Eleonora Vandini, Antonietta Vilella, Lars Dähne, Daniela Giuliani and Giuseppe Barillaro**

Supporting Information

Bioresorbable Nanostructured Chemical Sensor for Monitoring of pH Level In Vivo

Martina Corsi[1+], Alessandro Paghi[1+], Stefano Mariani[1], Giulia Golinelli[2], Aline Debrassi[3], Gabriella Egri[3], Giuseppina Leo[4], Eleonora Vandini[4], Antonietta Vilella[4], Lars Dähne[3], Daniela Giuliani[4] and Giuseppe Barillaro[1*]

[1]Dipartimento di Ingegneria dell'Informazione, Università di Pisa, via G. Caruso 16, 56122 Pisa, Italy
[2]Department of Medical and Surgical Sciences for Children & Adults, University-Hospital of Modena and Reggio Emilia, Via del Pozzo 71, 41124, Modena, Italy
[3]Surflay Nanotec GmbH, Max-Planck-Straße 3, 12489 Berlin, Germany
[4] Department of Biomedical, metabolic and Neural Sciences, University of Modena and Reggio Emilia, via G. Campi 287, 41125, Modena Italy
[+]These authors contributed equally to this work.
[*]Corresponding author: giuseppe.barillaro@unipi.it

Experimental Section

Materials and chemicals

Single side polished silicon boron-doped wafers (p^{++} type) with resistivity of 0.8 - 1.2 mΩ×cm, orientation <100>, were purchased from Siltronix Silicon Technologies (France). Single side polished silicon wafers (n type) with resistivity of 1 - 20 mΩ×cm, orientation <100>, were purchased from Siltronix Semirep (Germany). Aqueous hydrofluoric acid (HF, 48%), sodium hydroxide (NaOH, 98%), aqueous hydrochloric acid (HCl, 37%), sodium acetate (CH_3COONa, 99%), 4-(2-hydroxyethyl)-1-piperazineethanesulfonic acid (HEPES, 99.5%), sodium phosphate monobasic monohydrate (PBS, 98%), tris(hydroxymethyl)aminomethane (TRIS, 99%), sodium chloride (NaCl, 99%), chloroform ($CHCl_3$, > 99%), poly(D,L-lactide-co-glycolide) (PLGA, 85:15 lactide:glycolide, M_w = 50,000-75,000), paraformaldehyde (4%), glycerol, human serum albumin (fraction V), α-globulins Cohn (fraction IV-4) human, and ProClin™ 150 were purchased from Sigma Aldrich (Germany). Absolute ethanol (EtOH, 99.9%), isopropyl alcohol (IPA, 99.5%), and diethyl ether

(Et_2O, > 99%), were purchased from Carlo Erba Reagents (Italy). Rhodamine labelled poly-allylamine-hydrochloride (PAH:Rh (1:126)) and rhodamine labelled poly-methacrylic-acid (PMAA:Rh (1:164), M_w = 100,000) were provided by Surflay (Germany). 2 N skin toughness synthetic human tissues were purchased from SynDaver (USA). 10 mL polypropylene (PP) sterile syringes from PIC solution were purchased from Farmacia Raimo (Italy). PDMS (polydimethylsiloxane, Sylgard 184) base and thermal curing agent were purchased from Cecchi S.r.l. (Italy). Aqueous solutions were prepared using deionized water (DIW, 15 MΩ×cm) filtered by Elix®, Merck Millipore (Germany). All buffers were prepared in deionized water (DIW) and pH-adjusted with NaOH (5 M) and HCl (1 M) aqueous solution. General anesthetic isoflurane was purchased from 2Biol (Italy). Ketamine and xylaxine were purchased from veterinary pharmacy "la veterinaria" Pisa (Italy). Betadine and neuflan were purchased from hospital pharmacy of Modena (Italy); eosin and hematoxylin from Histo-Line lab (Italy).

Fabrication of nanostructured porous silicon oxide (nPSiO$_2$) scaffolds on PLGA foil

Nanostructured porous silicon (nPSi) layers were prepared by room temperature (RT) anodic etching of p-type silicon (1.5 × 1.5 cm^2) using 2 mL of a HF:EtOH (3:1 v/v) solution. A custom-made Teflon cell equipped with a platinum wire cathode and an aluminum flat anode, namely, two-electrode electrochemical cell, was used for the electrochemical anodization of the silicon substrate over a circular area of 0.567 cm^2. A source measurement unit (SMU 2602A, Keithley, USA) was used to set the etching current and measure the voltage between anode and cathode.

A first sacrificial nPSi layer was etched at 500 mA cm^{-2} for 15 s, rinsed with EtOH for 120 s to remove residual HF, and fully dissolved in NaOH(1M):EtOH (9:1 v/v) solution for 120 s to achieve a nanostructured templating surface that avoid formation of a parasitic layer and increase pore size of the next-etched nPSi layer[1]. The so-processed sample was rinsed in DIW and EtOH and used to etch a second nPSi layer at 5 mA cm^{-2} for 40 s with thickness of ~100 nm, porosity of ~61.5 %, and pore

size about ~7 nm, namely, barrier layer, to inhibit diffusion of PLGA inside the pore structure after the transfer-printing process[2]. The sample was rinsed in EtOH for 120 s to remove residual HF, then a third nPSi layer with thickness of ~4.3 μm thickness and porosity of ~77 %, namely, functional layer, was etched at 500 mA cm^{-2} for 25 s under the barrier layer. The sample was rinsed in EtOH for 120 s and Et_2O for 60 s to obtain a crack-free nPSi layer.

The as-prepared nPSi stack was transfer-printed on a flexible PLGA foil as reported by Wan *et al.*[3]. First, the nPSi edge was scratched with a diamond tip to separate the porous layer from lateral bulk silicon. The so-scratched sample was inserted again in the electrochemical cell and the nPSi stack was fully detached from the Si substrate underneath through electropolishing of crystalline silicon at the bottom of the nPSi stack, achieved at 800 mA cm^{-2} for 0.1 s using 1 mL of a HF:EtOH (1:1 v/v) solution. The HF concentration of the solution was reduced with respect to that used for the etching of the nPSi stack to lower the electropolishing current[4]. The free-standing nPSi membrane was gently rinsed in EtOH and in Et_2O, then glided (barrier layer on top) onto the non-polished side of a silicon chip and topped with a second non-polished silicon chip (in contact with the barrier layer). The nPSi membrane sandwiched between the two silicon chips was oxidized to $nPSiO_2$ at 1000 °C for 5 min in a muffle furnace (ZB/1, ASAL, Italy). The silicon chip on top was lifted and a PLGA foil (3 cm × 2 cm, thickness of 40 μm) was placed on top of the as-prepared $nPSiO_2$ membrane, in contact with the barrier layer. The PLGA-$nPSiO_2$-Si assembly was heated at 100 °C for 60 s onto a hotplate (ARE 230V, Velp Scientifica, Italy), then rinsed with DIW to detach the PLGA-$nPSiO_2$ assembly from the Si chip. The $nPSiO_2$ membrane transfer-printed on the PLGA foil was eventually gently dried at 30 °C for 5 min in a ventilated oven (G-Therm 035, Fratelli Galli, Italy).

Preparation of PLGA foil

A PLGA solution was prepared dissolving 0.2 g of PLGA in 10 mL of chloroform at RT, then stirring the solution gently for 6 hours under a glass bell to limit solvent evaporation. The as-prepared solution was dropped on the surface of a SiO_2/Si wafer (SiO_2 thickness ~100 nm, wafer diameter 7 cm) previously treated with an oxygen plasma (Smart Plasma, PLASMA Technology) at 80 W for 5 min to increase the surface hydrophilicity to facilitate PLGA peeling-off. The PLGA-coated wafer was left overnight under a glass bell saturated with chloroform vapors, then vacuumed for 2 hours using a rotation pump to fully remove the solvent from the PLGA foil. Eventually, the PLGA foil was peeled off the SiO_2/Si wafer upon immersion of the wafer in DIW to facilitate the film detachment. The PLGA foil was eventually gently dried at 30 °C for 5 min in a ventilated oven (G-Therm 035, Fratelli Galli, Italy).

Fabrication of nPSiO$_2$ scaffolds on silicon chips

Nanostructured porous silicon (nPSi) layers standing on silicon chips were prepared by room temperature (RT) anodic etching of p-type silicon (1.5 × 1.5 cm^2) using 2 mL of a HF:EtOH (3:1 v/v) solution. A custom-made Teflon cell with a platinum wire cathode and an aluminum flat anode, namely, two-electrode electrochemical cell, was used for electrochemical anodization of the silicon substrate over a circular area of 0.567 cm^2. A source measurement unit (SMU 2602A, Keithley, USA) was used to set the etching current and measure the voltage between anode and cathode.

A first sacrificial nPSi layer was etched at 500 mA cm^{-2} for 15 s, rinsed with EtOH for 120 s to remove residual HF, and fully dissolved in NaOH(1M):EtOH (9:1 v/v) solution for 120 s to achieve a nanostructured templating surface that avoid formation of a parasitic layer and increase pore size of the next-etched nPSi layer[1].

In a first set of experiments, the above-processed sample was rinsed in DIW and EtOH and used to etch a second nPSi layer at 500 mA cm^{-2} for 25 s with thickness of ~4.3 µm and porosity of ~77 %.

Then, the sample was rinsed in EtOH for 120 s and Et_2O for 60 s to obtain a crack-free nPSi layer. Eventually, nPSi layers were thermally oxidized to $nPSiO_2$ in a muffle furnace (ZB/1, ASAL, Italy) at different temperatures for different times, namely 500 °C for 30 min, 750 °C for 30 min, and 1000 °C for 5 min.

In a second set of experiments, the above-processed sample was rinsed in DIW and EtOH and used to etch a second nPSi layer at 500 mA cm^{-2} for 12 s, 25 s, and 50 s to fabricate nPSi layer with porosity of about 77% and thickness of about 2 μm, 4 μm, and 8 μm, respectively. Then, samples were rinsed in EtOH for 120 s and Et_2O for 60 s to obtain a crack-free nPSi layer. Eventually, nPSi layers were thermally oxidized to $nPSiO_2$ in a muffle furnace (ZB/1, ASAL, Italy) at 1000 °C for 5 min.

Coupling reaction of Rhodamine-B with polyelectrolytes

The Rhodamine-B derivative was synthetized with a linker containing an amine group (Rh-amine). The side chain containing the amine group was attached via an amide bond. For negatively-charged polyelectrolytes, namely, poly-methacrylic-acid (PMAA), the carboxylic group was activated by N-(3-Dimethylaminopropyl)-N′-ethylcarbodiimide hydrochloride (EDAC) or 1,1'-Carbonyldiimidazole (CDI), and the Rh-amine was coupled by amide bond. For positively-charged polyelectrolytes, namely, poly-allylamine-hydrochloride (PAH), the Rh-amine was converted to an isothiocyanate, which reacted with the amine groups of the PAH.

Layer-by-layer coating of $nPSiO_2$ scaffolds with fluorescent polyelectrolytes

$nPSiO_2$ scaffolds on PLGA foil and bulk silicon were coated with a pH responsive polyelectrolyte stack leveraging a layer-by-layer (LbL) electrostatic assembling, as previously reported by our group[5,6]. Specifically, the optimized pH sensor was achieved as follows: 1) PAH:Rh (1 mg mL^{-1} in 50 mM TRIS buffer with 200 mM NaCl at pH 8) was used as the positively-charged polyelectrolyte.

The PAH:Rh solution (50 μL) was drop cast on top of the $nPSiO_2$ scaffold and incubated for 1 hour at RT. 2) PMAA:Rh (1 mg mL^{-1} in 50 mM TRIS buffer with 200 mM NaCl at pH 8) was used as the negatively-charged polyelectrolyte. The PMAA:Rh solution (50 μL) was drop cast on top of the PAH:Rh-coated $nPSiO_2$ scaffold and incubated for 1 hour at RT. After each coating step, the scaffold on PLGA foil was rinsed with EtOH for 5 s, DIW for 1 min, and eventually gently dried at 30 °C for 5 min in a ventilated oven (G-Therm 035, Fratelli Galli, Italy), whereas the scaffold on silicon chip was rinsed with DIW and dried under a nitrogen flow.

Coating steps 1) and 2) were repeated to change the number of polyelectrolytes of the polymer stack assembled within the $nPSiO_2$ scaffold, namely, from $(PAH{:}Rh/PMAA{:}Rh)_1$ to $(PAH{:}Rh/PMAA{:}Rh)_3$+$(PAH{:}Rh)_1$.

Morphological characterization of nPSi and $nPSiO_2$ scaffolds

Morphological characterization of nPSi layers (top-view and cross-section) was carried out using a scanning electron microscope (FEG-SEM, Zeiss SUPRA) with a 10 kV acceleration voltage at different magnifications. Distribution of the pore diameters was obtained from the analysis of top-view SEM image with Gwyddion software. Coating of $nPSiO_2$ scaffolds with PAH:Rh and PMAA:Rh was investigated in cross-section both in bright-field and fluorescence mode with an optical microscope (Leica DM2500 M) equipped with a green laser (CPS520, λ_{ex} = 520 nm, 4.5 mW, 4.6 × 1.7 mm^2 beam shape, Thorlabs, USA) as the excitation source.

Optical characterization of nPSi and $nPSiO_2$ scaffolds

Reflectance spectrum of bare and polymer-coated nPSi and $nPSiO_2$ scaffolds on silicon chip was measured in the wavelength range 400 - 1000 nm at normal incidence in air (for dry samples) or through a sapphire window (for wet samples secured within a flow cell). Light exiting the source

(Deuterium tungsten halogen light source DH-2000-BAL, OceanOptics, USA) was fed through one arm of a bifurcated fibre-optic probe (QR200-7-SR, OceanOptics, USA) orthogonally onto the sample surface, and the reflected light was collected through the other arm into a UV-VIS spectrometer (USB2000+UV-VIS, Ocean Optics, USA). Acquisition parameters, namely, integration time, average scan number, and boxcar width, were: 2 ms, 2, and 5 for measures carried out in air on dry samples; 10 ms, 10, and 1 for measures carried out through the sapphire window of a flow cell on wet samples, with the spectrometer working normalized reflectance mode.

Porosity and thickness of the porous scaffolds were evaluated by best-fitting of reflectance spectra of the as-prepared nPSi layers with a homemade Matlab software (MathWorks, USA)[8].

Fast Fourier Transform Reflectance Spectroscopy (FFT-RS, Hanning window) of the reflectance spectra of the porous scaffolds was used to calculate the Effective Optical Thickness (EOT) = $2n_{eff}$ L value, with L thickness and n_{eff} effective refractive index of the scaffolds.

Photoluminescence measurements of nPSiO$_2$ scaffolds coated with fluorescent polyelectrolytes

Photoluminescence spectra of nPSiO$_2$ scaffolds on PLGA foil and bulk silicon and control SiO_2 flat substrates coated with polyelectrolytes were acquired in the wavelength range 400 - 1000 nm in air or through a sapphire window for samples secured within a flow cell. The excitation source was a green laser diode (CPS520, λ_{ex} = 520 nm, 4.5 mW, 4.6 × 1.7 mm^2 beam shape, Thorlabs, USA) focused on the sample with an incident angle of ~30° to the surface, and the reflected light was collected through one arm of a bifurcated fibre-optic probe (QR200-7-SR, OceanOptics, USA) into a UV-VIS spectrometer (USB2000+UV-VIS, Ocean Optics, USA). Acquisition parameters, namely, integration time, average scan number, and boxcar width, were: 1s, 2, and 5 for measures carried out in air; 2s, 2, and 5 for measures carried out through a sapphire window on samples within the flow cell, with the spectrometer working in scope mode.

Measurement of the thickness of PAH:Rh/PMAA:Rh polyelectrolyte stacks

Ellipsometry measurements were performed on flat SiO_2(110 nm)/Si samples (2 cm × 1.5 cm) coated with PAH:Rh and PMAA:Rh polyelectrolytes using an ellipsometer (RudolphResearch/AutoEL-II Automatic Ellipsometer) at an excitation wavelength of 633 nm and 70° incidence angle. Coating of the samples with the polyelectrolyte stack was carried out according to the following procedure: 1) the SiO_2/Si samples were dipped in 10 mL of PAH:Rh solution at RT for 120 s, washed in 40 mL of TRIS buffer pH 8 for 30 s to remove the excess of polymer, and dried with N_2; 2) the PAH:Rh-coated samples were dipped in 10 mL of PMAA:Rh solution at RT for 120 s, washed in 40 mL of TRIS buffer pH 8 for 30 s, and eventually dried with N_2. The steps 1) and 2) were repeated as needed to increase the number of polyelectrolytes in the assembled stack. The number of PAH:Rh and PMAA:Rh layers was varied from $(PAH:Rh)_1$ to $(PAH:Rh/PMAA:Rh)_3+(PAH:Rh)_1$.

Thickness of the polyelectrolyte stack at each step was estimated by best-fitting experimental raw Delta (Δ) and Psi (Ψ) ellipsometry values using a customized homemade Matlab software (MathWorks, USA)[8].

Optimization of pH sensitivity of the fluorescent polymer stack

pH sensitivity of $nPSiO_2$ scaffolds coated with fluorescent polyelectrolytes was investigated in different assembly buffers and for different polymer stack architectures.

As to the assembly buffer, the Rh photoluminescence emission increased with the number of polyelectrolytes assembled in the $nPSiO_2$ scaffolds regardless of the buffer used, achieving a maximum intensity in acetate buffer. On the other hand, photoluminescence changes to pH (i.e., sensitivity) of the polymer stack assembled in the different buffers were appreciably higher in TRIS buffer at pH 8 (i.e., 6112 counts/pH) than in TRIS at pH 7 (i.e., 3616 counts/pH) and acetate (i.e.,

4591 counts/pH). Therefore, TRIS buffer at pH 8 was chosen as the assembly buffer for all the next experiments.

As to the polymer stack architecture, different configurations of the polymer stack were tested, i.e., $(PAH:Rh/PMAA:Rh)_x$ and $(PAH:Rh/PMAA:Rh)_x+(PAH:Rh)_1$ with *x* ranging from 1 to 3, and higher sensitivity was achieved for polymer stack with an odd number of layers. In fact, the LbL assembling leads to electroneutrality inside the film at the pH of assembling, but the outmost layer always results in an excess of charges. For an even stack, i.e., $(PAH:Rh/PMAA:Rh)_{x,}$ PMAA is the outermost layer, thus there is an excess of negative charges. The ratio of excess charges in respect to the total assembled material decreases with each additional pair of PAH/PMAA layers. In the odd stack, i.e., $(PAH:Rh/PMAA:Rh)_x+(PAH:Rh)_1$, PAH is the outermost layer and a remarkably higher excess of total charges than the even case is established. With decreasing pH, with respect to pH of assembling, the increasing positive charges in the stack, especially in case of odd number of polymers, leads to a collection of negatively-charged counterions (ions Cl^- or $H_2PO_4^-$ of PBS buffer) that regulate the swelling of the polymer stack and, in turn, the increase of the photoluminescence.

Preparation of synthetic interstitial fluid solution

Synthetic interstitial fluid (ISF) solutions containing 11 gL^{-1} of total proteins were prepared by dissolving human albumin (fraction V) and α-globulins (Cohn fraction IV-1) in a ratio 60:40 in standard physiological solution (0.9% NaCl), containing 6 mgL^{-1} of ProClin™ 150 as preservative.

In-vitro pH sensing experiments

pH sensing with polymer-coated $nPSiO_2$ scaffolds, both on PLGA foil and silicon chips, was carried out either using a Petri dish or securing the sensor within a homemade flow-cell, at a constant temperature of 37 °C in a ventilated oven (G-Therm 035, Fratelli Galli, Italy). The photoluminescence

spectrum was acquired as detailed in section *Photoluminescence measurement of $nPSiO_2$ scaffolds coated with fluorescent polyelectrolytes*, after immersion of the sensor in a PBS solution (10mM PBS with 100mM NaCl) or ISF solution with different pH values in the range 4 – 7.5.

As to the flow-cell system, the PBS solution was injected in the flow-cell using a syringe pump (Nexus 3000, Chemyx Inc., USA), according to the following two-phase protocol that applies to each different pH value: 1) injection for 4 min at a flow rate of 100 μL min^{-1} to ensure the filling up of the system with the solution; 2) injection for 30 min at a flow rate of 20 μL min^{-1} to ensure a steady-state photoluminescence value is reached. Photoluminescence spectra were acquired at the end of phase 2) for steady-state measurements aimed at retrieving the calibration curve of the sensor; the spectra were acquired every 5 min from beginning to end of phase 2) for time-resolved measurements aimed at evaluating the sensor kinetics. Acquisition parameters were: integration time 2s, average scan number 2, and boxcar width 5, with the spectrometer working in scope mode.

As to experiments in Petri dish, the sensor was placed for 30 min in a PS Petri dish (3.5 cm in diameter) containing 5 mL of a PBS solution with a given pH value. The sensor was then taken out of the Petri dish and the photoluminescent spectrum was acquired. The sensor was then rinsed in DIW for 1 min to eliminate residual of the precedent buffer and placed in the Petri dish filled with a PBS solution with a different pH value. The procedure was repeated for all the pH values to be tested in the range 4 – 7.5. Acquisition parameters were: integration time 1s, average scan number 2, and boxcar width 5, with the spectrometer working in scope mode.

Evaluation of intrinsic sensitivity of PAH:Rh and PMAA:Rh to pH

PAH:Rh and PMAA:Rh solutions (600 μL of 0.05 mg mL^{-1} in 10 mM PBS buffer with 100 mM NaCl) with different pH values, namely, 4, 5, 6, and 7, were prepared and dropped into a quartz μ-fluorescence Cuvette (CV10Q700, 700 μL volume, Thorlabs, USA) for photoluminescence spectra

measurement. The excitation source was a green laser diode (CPS520, λ_{ex} = 520 nm, 4.5 mW, 4.6 × 1.7 mm^2 beam shape, Thorlabs, USA) focused on the cuvette, and the 90° emitted light was collected through an optical fibre (QR600-7-VIS-NIR, OceanOptics, USA) into a UV-VIS spectrometer (USB2000+UV-VIS, Ocean Optics, USA). Acquisition parameters were: integration time 400 ms, average scan number 5, boxcar width 1, with the spectrometer working in scope mode.

pH sensing using synthetic skin

A synthetic skin sample (SynDaver, 3 cm × 2 cm, 1.5-mm-thick, bi-layer) was soaked overnight in 40 mL of a PBS solution (10mM PBS with 100mM NaCl) at pH 7.4. The pH sensor (i.e., a polymer-coated $nPSiO_2$ scaffold on PLGA foil) was sandwiched between the two layers of the soaked skin and its photoluminescence spectrum acquired through the skin to verify stability of the photoluminescence intensity for 30 min. Next, alternate injections (3 repeats) of 2 mL (4 x 500 μL) of a PBS solution with pH of 4 or 7.4 were performed around the sensor site to induce a local change of the skin pH every 90 min. After each injection, the photoluminescence spectrum of the sensor was acquired through the skin for 90 min to investigate changes of the sensor photoluminescence intensity with pH before injection of solution with different pH.

The photoluminescence spectrum was acquired using the system detailed in section *Photoluminescence measurement of $nPSiO_2$ scaffolds coated with fluorescent polyelectrolytes*, Acquisition parameters were: integration time 2s, average scan number 2, and boxcar width 5. Photoluminescence spectra were acquired every 5 min.

pH sensing using a light source-photodiode pair

pH sensing with polymer-coated $nPSiO_2$ scaffolds was carried out in a Petri dish at a constant temperature of 37 °C in a ventilated oven (G-Therm 035, Fratelli Galli, Italy). The sensor was placed

for 30 min in a PS Petri dish (3.5 cm in diameter) containing 5 mL of a PBS solution with a given pH value. The sensor was then taken out of the Petri dish and the photoluminescent spectrum was acquired. The sensor was then rinsed in DIW for 1 min to eliminate residual of the precedent buffer and placed in the Petri dish filled with a PBS solution (10mM PBS with 100mM NaCl) with a different pH values. The procedure was repeated for all the pH values to be tested in the range 4 – 7.5.

Photoluminescence intensity of polymer-coated $nPSiO_2$ scaffolds at different pH values was measured in air with a high precision photodiode with dimension 5 mm × 3 mm × 2.1 mm and peak sensitivity of 560 nm (SFH 2270R, OSRAM, Germany). The sensor photoluminescence was collected through one arm of a bifurcated fibre-optic probe (QR200-7-SR, OceanOptics, USA) and redirected to the photodiode using an optical fiber (M35L01, Thorlabs, USA) with an in-line long-pass filter with cut-off wavelength of 515 nm (GL-OG515-3-12, Optoprim, Italy). A green laser diode (CPS520, λ_{ex} = 520 nm, 4.5 mW, 4.6 × 1.7 mm^2 beam shape, Thorlabs, USA) focused on the sample with an incident angle of ~30° to the surface was used as the excitation source. The photodiode was powered with -1 V, generated with a buffered resistive voltage divider from a voltage source (E3631A, Keysight, USA) of -3.5 V. The photocurrent I_{FD} is converted into the voltage V_{TIA} by means of a transimpedance amplifier (TIA) and next amplified by a factor 101 using a non-inverting amplifier, thus obtaining the output voltage V_{OUT}. (Figure S12)

In-vitro dissolution of pH sensor in flow cell

Dissolution of $nPSiO_2$ scaffolds on silicon chips oxidized at 1000 °C for 5 min and coated with $(PAH:Rh/PMAA:Rh)_2$+$(PAH:Rh)_1$ was carried out in a flow cell to simulate physiological interstitial fluid flow. The $nPSiO_2$ scaffolds were secured into a flow cell system placed in a ventilated oven at 37°C (G-Therm 035, Fratelli Galli, Italy). A syringe-pump (Nexus 3000, Chemyx Inc., USA) was used to inject a HEPES buffer solution (10 mM HEPES with 100 mM NaCl at pH 7.4) at a flow rate of 100 µL min^{-1} for 4 min to fill up the system. HEPES buffer was then injected in the flow cell at a

flow rate of 0.7 μL min^{-1} and reflectance and photoluminescence spectra were acquired as detailed in section *Optical characterization of nPSi and nPSiO$_2$ scaffolds by reflectance spectroscopy* and *Photoluminescence measurement of nPSiO$_2$ scaffolds coated with fluorescent polyelectrolytes*, respectively. Peak amplitude and position of the FFT spectrum were used as dissolution indicators[9], as also confirmed by theoretical calculation based on Transfer Matrix Method (TMM) and leveraging a 3-components Bruggeman equivalent medium approximation for the nPSiO$_2$ scaffold, carried out using a homemade Matlab software (MathWorks, USA). Reflectance and photoluminescence spectra were acquired every hour until FFT and PL peak values reduced to those of the noise floor, namely, 3.3σ_0, with σ_0 standard deviation of the background signals. At the end of the experiment, the surface of the silicon substrate at the pH sensor location was investigated for possible polymer residues.

Dissolution of bare nPSiO$_2$ scaffolds on silicon chips oxidized in different conditions (control samples) was carried out in a PS Petri dish (9 cm in diameter) containing 40 mL of a HEPES buffer solution (10 mM HEPES with 100 mM NaCl at pH 7.4) at 37 °C (Unitemp, HP-155, Germany). Dissolution of the nPSiO$_2$ scaffolds over time was monitored through acquisition of nPSiO$_2$ reflectance spectrum every hour and successive calculation of the FFT spectrum, as detailed in section *Optical characterization of nPSi and nPSiO$_2$ scaffolds by reflectance spectroscopy*. Reflectance spectra were acquired every hour until the FFT peak value reduced to that of the noise floor, namely, 3.3σ_0, with σ_0 is the standard deviation of the background signal.

In-vitro dissolution of pH sensor on PLGA foil under synthetic skin

A synthetic skin sample (SynDaver, 3 cm × 2 cm, 1.5-mm-thick, bi-layer) was soaked overnight in a Petri dish filled with 40 mL of a PBS solution (10mM PBS with 100mM NaCl) at pH 7.4. The pH sensor on PLGA foil was sandwiched between the two flaps of the soaked skin, leaving the skin with the pH sensor in between immersed the PBS solution at pH 7.4 until the end of the experiment. Photoluminescence emission of the pH sensor was acquired through the skin using the system detailed

in section *Photoluminescence measurement of nPSiO$_2$ scaffolds coated with fluorescent polyelectrolytes.* Acquisition parameters were: integration time 2s, average scan number 2, and boxcar width 5. Specifically, the skin incorporating the pH sensor was taken out of the Petri dish for 30 s every 4 hours to measure the photoluminescence spectrum in air, then put back into the PBS solution. The measurements were carried out until the PL peak value reduced to that of the noise floor, namely, 3.3σ0, with σ0 is the standard deviation of the background signal. At the end of the experiment the two flaps of the skin were taken apart and the inner surface of the skin flaps was investigated for possible macroscopic polymer residues at the sensor location, through both optical investigation and PL measurement.

Animal source

Adult male and female mice (n= 14) of the C57BL6 /J strain were kept separated by sex in air-conditioned colony rooms (temperature 21 ± 1 °C, humidity 60%) on a natural light/dark cycle, housed in groups (and individually during the experimental phase) with access to food (without chlorophyll, to reduce autofluorescence during device monitoring) and water ad libitum, always with structural enrichments in their cages.

Housing conditions and experimental procedures were in strict accordance with the "Principles of laboratory animal care"[10] and the European Community regulations on the use and care of animals for scientific purposes[11], and were approved by the Committee on Animal Health and Care of Modena and Reggio Emilia University.

Sensor implantation. The mice (n=9) were anaesthetized in an induction chamber with 4% isoflurane (2Biol, Italy) and maintained at 2% using a vaporizer with 0.4 L min^{-1} of O_2 during the surgical act. The state of deep anaesthesia with isoflurane was assessed (slowing of respiratory rate and lack of

reflex responses). The back of the mice was shaved and disinfected with 10% Betadine skin solution; an incision of 1 cm was made with a sterile scalpel in the skin on the central part of the back, a pocket was created and the pH sensor on PLGA foil (0.8 cm × 0.8 cm) was inserted in the subcutis. The incision was sutured with surgical stitch and treated with Neuflan gel (Neomycin 0.5 g / Flucinolone acetonide 0.025 g / Lidocaine 2.5 g) to minimize local post-operative pain and infection risks. Control mice (n=5) were subjected to the same surgical procedure except for device implantation.

Each mouse was kept under continuous observation until complete awakening from anaesthesia, then placed in the cage individually to reduce the risk that cage mates could interfere with each other on wounds, compromising the device stability**.**

Bioabsorption analysis. At endpoints 7, 14, 21 and 60 days after implantation of the sensor, a group of mice (n=6) was anesthetized with isoflurane. Bioabsorption of the sensors was verified by collecting fluorescence images at each endpoints using an IVIS Lumina XRMS in-vivo animal imager (PerkinElmer Inc, Waltham, MA). The instrument was set up with 460-560 nm excitation and 620 nm emission filters. Autofluorescence was adjusted using guided unmixing procedures available through IVIS software (Living Image Software, PerkinElmer Inc). Fluorescence intensity of at the implant site was evaluated using a circular ROI with constant diameter (for all mice and images) and represented with the total radiance efficiency calculated as the radiant efficiency per incident excitation summed over the number of pixels of the ROI. All mice were weighed before surgical implant of the sensor and after 60 days.

In-vivo pH sensing. Another group of mice (n=3) was anesthetized with a longer-acting anesthesia of isofluorane ketamine (100 mg kg^{-1}) and xylazine (20 mg kg^{-1}) intraperitoneally (IP). Injections of 2 mL (4 x 500 μL) of a PBS solution were performed around the sensor site to induce a local change of

the skin pH at 30 min and 60 min with pH 4, and then 90 min with pH 7.4, using the same protocol validated for pH sensing under synthetic skin detailed in section "*pH sensing using synthetic skin*". Fluorescence images were collected using an IVIS Lumina XRMS in-vivo animal imager (PerkinElmer Inc, Waltham, MA) every 10 min until 2 hours from implant. The instrument was set up with 460-560 nm excitation and 620 nm emission filters. Autofluorescence was adjusted using guided unmixing procedures available through IVIS software (Living Image Software, PerkinElmer Inc). Fluorescence intensity of at the implant site was evaluated using a circular ROI with constant diameter (for all mice and images) and represented with the total radiance efficiency calculated as the radiant efficiency per incident excitation summed over the number of pixels of the ROI.

Biocompatibility analysis

After 60 days from pH sensor implantation, mice (n=6) were anesthetized with isoflurane and sacrificed for histological processing to verify biocompatibility of the sensor following the guidelines of Annex E of UNI EN ISO10993-6, namely, macroscopic evaluation of the surgical area using a 4-point scale (i.e., 0=intact skin; 1=swelling, 2=redness, 3=eschar) and microscopic evaluation of square skin area samples adjacent to the implant site (1.5 × 1.5 cm^2) and organs (liver, kidney, heart, brain, lung and spleen) through histological investigations. Fluorescence images of whole organs were collected with IVIS Lumina XRMS in-vivo animal imager (PerkinElmer Inc, Waltham, MA) using 520 nm excitation and 620 nm emission filters. Fluorescence intensity of each organ was evaluated using a manual ROI and represented with the average radiance efficiency calculated as the radiant efficiency per incident excitation normalized to the number of pixels of the ROI. Immediately after this detection, the samples (i.e., skin and organs) were postfixed in the paraformaldehyde solution (4%) for 12 hours, rinsed in a 30% sucrose-PBS solution for two days. The skin was then frozen using dry ice, and coronal 40 μm thick sections series were cut at a cryotome, washed three times in cold PBS and stored at -20 °C in a glycerol-PBS solution until use. On samples a

hematoxylin-eosin dichromatic coloring was carried out. Each preparation was subjected to morphological evaluation by optical microscopy. The taken parameters were histological architecture, nucleus degeneration, nuclear dust, pyknosis, cellular shrinkage, pericellular vacuolization.

Statistical Analysis

The results are presented as mean with standard deviation ($n \geq 3$) values. All the data were subjected to two-tailed Student's t-test for statistic comparisons using Matlab Software. In all cases, significance was defined as $p \leq 0.01$ and symbols were used to indicate statistically independent values.

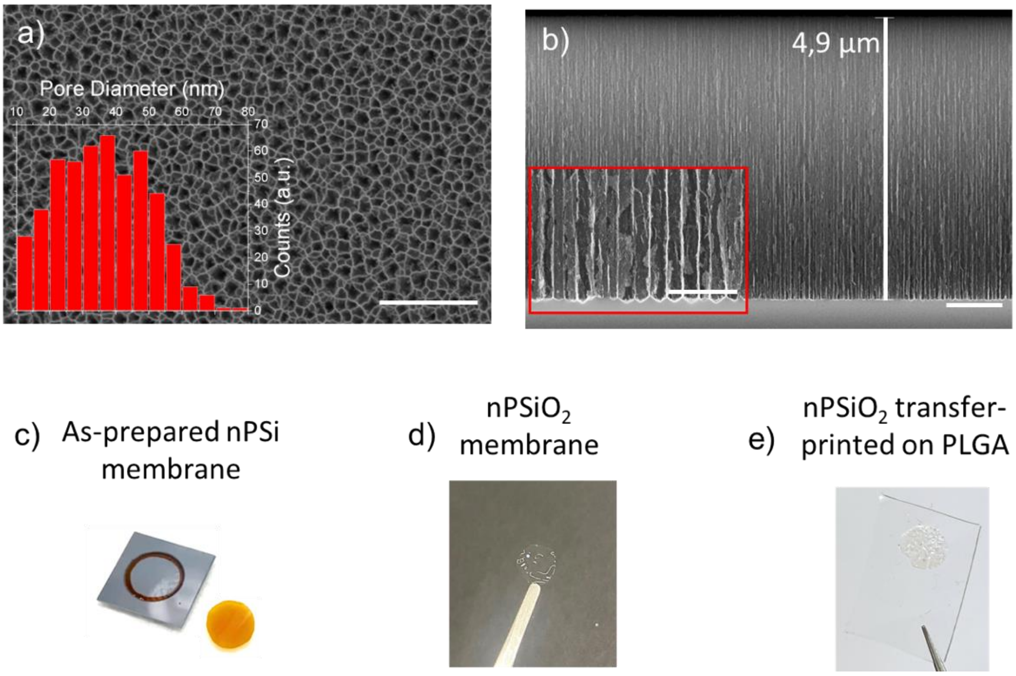

Figure S1. **Images of as-prepared nPSi scaffold, oxidation to nPSiO$_2$, and transfer-printing on PLGA foil.** a) Top-view SEM image (magnification 250000×) of a nPSi scaffold with porosity of ~77 %. Scale bar is 400 nm. Inset shows the histogram of the size distribution of pores with average diameter of 35 nm. b) Cross-section SEM image (magnification 35000×) of the nPSi scaffold in a). Scale bar is 1 µm. Inset shows a magnification (250,000×) of the bottom part of the scaffold that allows to better appreciate the columnar morphology of the pores. Scale bar is 400 nm. c) Picture of a 5-µm-thick nPSi membrane lifted-off from the native bulk silicon. d) Picture of the nPSi membrane in c) oxidized to nPSiO$_2$ at 1000 °C for 5 min. e) Picture of nPSiO$_2$ membrane in d) after transfer-printing on a PLGA foil.

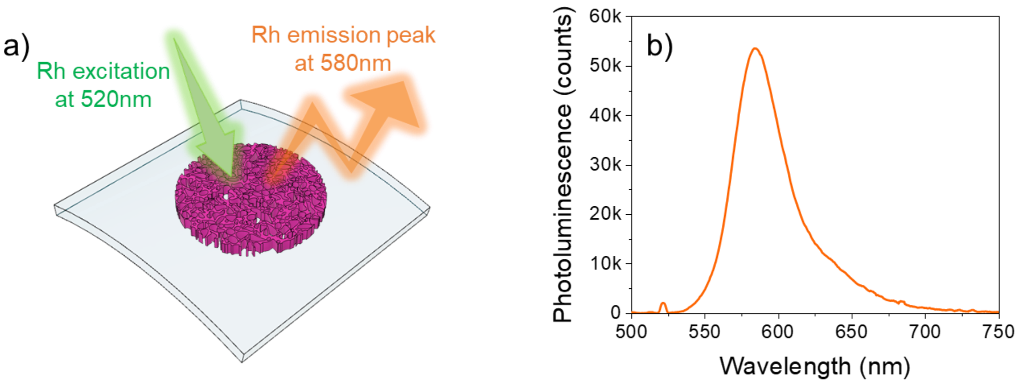

Figure S2. **Photoluminescence spectrum of a nPSiO$_2$ membrane coated with PAH:Rh and PMAA:Rh polyelectrolytes.** a) Sketch of a nPSiO$_2$ scaffold on PLGA foil coated with Rh-labelled polyelectrolytes via LbL technique. Rh excitation peak at 520 nm/emission peak at 580 nm. b) Photoluminescence spectrum of a nPSiO$_2$ scaffold on PLGA foil coated with (PAH:Rh/PMAA:Rh)$_2$+(PAH:Rh)$_1$.

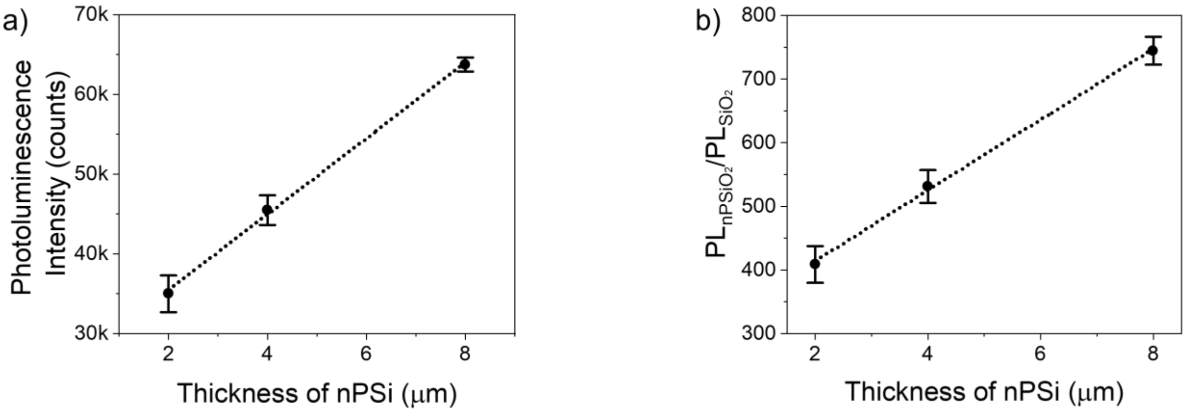

Figure S3. Effect of nPSi thickness on the fluorescent intensity of the polymer stack deposited within $nPSiO_2$ scaffolds. a) Photoluminescence intensity at 580 nm acquired on $nPSiO_2$ scaffolds with different thicknesses, namely, 2, 4, and 8 μm, coated with $(PAH:Rh/PMAA:Rh)_2$+$(PAH:Rh)_1$ (n=3 samples for each thickness). b) Photoluminescence intensity ratio (at 580 nm) between $nPSiO_2$ scaffolds and a flat $PSiO_2$ substrate (i.e., PL_{nSiO2} /PL_{SiO2}) coated with $(PAH:Rh/PMAA:Rh)_2$+$(PAH:Rh)_1$ at different $nPSiO_2$ thicknesses (n=3 samples for each thickness). Data are presented as mean (± s.d).

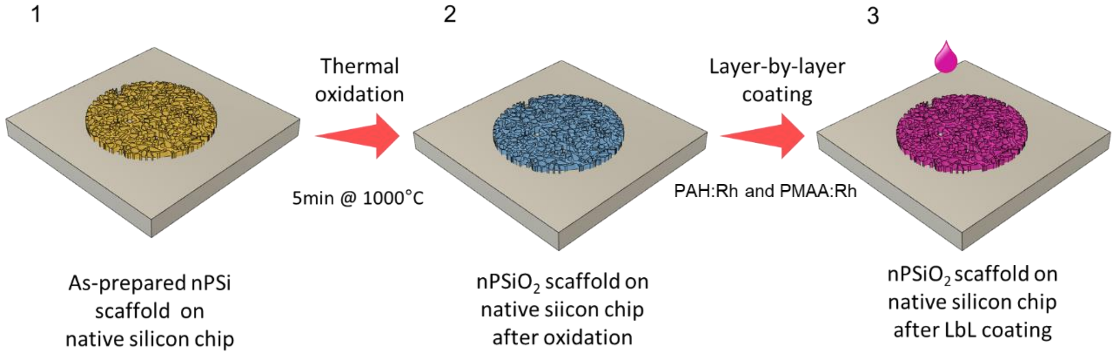

Figure S4. **Main fabrication steps of a $nPSiO_2$ scaffold coated with fluorescent polyelectrolytes on native silicon chip.** 1. Fabrication of the nPSi scaffold via electrochemical anodization of silicon substrate; 2. Thermal oxidation of nPSi to $nPSiO_2$ at 1000 °C for 5 min in a muffle furnace on the native silicon; 3. Coating of $nPSiO_2$ scaffold with Rh-labelled polyelectrolytes via LbL technique on the native silicon chip.

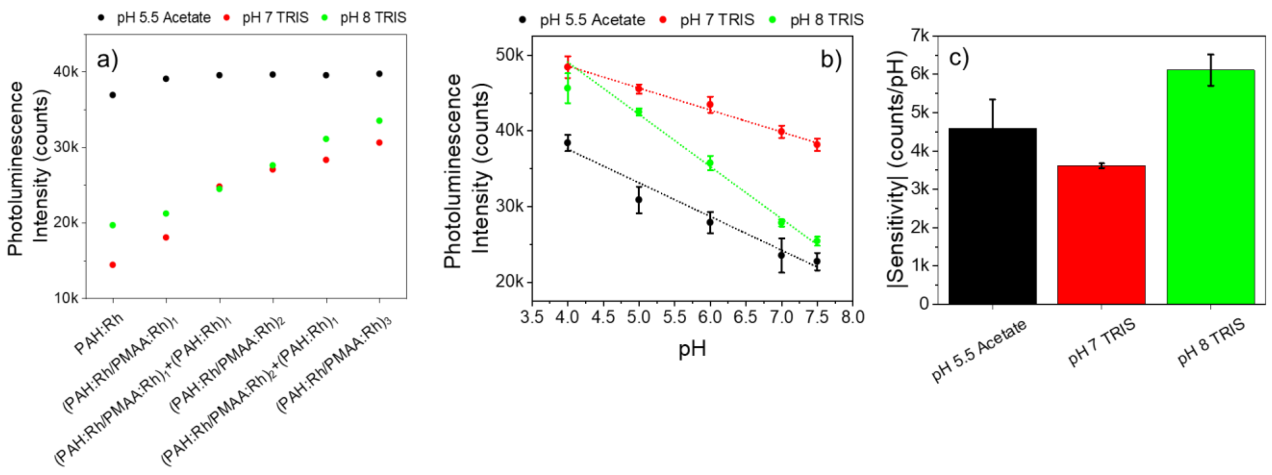

Figure S5. **Effect of the assembly buffer of the fluorescent polymer stack deposited within nPSiO$_2$ scaffolds on the pH sensing performance.** a) Photoluminescence intensity at 580 nm of nPSiO$_2$ scaffolds on silicon chip coated with a fluorescent polymer stack with a different number of layers using different assembly buffers, namely, acetate at pH 5.5, TRIS at pH 7, and TRIS at pH 8. b) Calibration curve (Photoluminescence intensity at 580 nm *vs.* pH value) of nPSiO$_2$ scaffolds on silicon chip coated with (PAH:Rh/PMAA:Rh)$_2$+(PAH:Rh)$_1$ in three different assembly buffers. The calibration curves are measured in PBS solution with different pH values in the range 4 – 7.5, regardless of the assembly buffer. (n=3 pH cycles) c) Sensitivity to pH of the sensors in b) (n=3 pH cycles). In b) and c) data are presented as mean (± s.d).

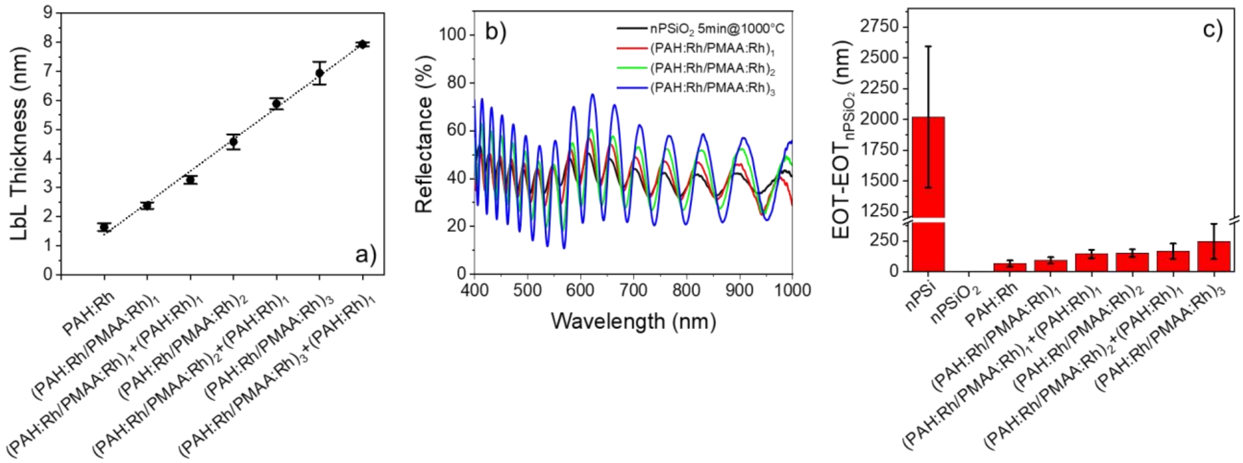

Figure S6. **Ellipsometry and optical characterizations of PAH:Rh/PMAA:Rh polyelectrolyte stacks.** a) Ellipsometry measurements of the thickness of PAH:Rh and PMAA:Rh polyelectrolytes assembled as a multilayer on a flat SiO$_2$(110nm)/Si sample (n=3 scaffolds for each polyelectrolyte architecture). b) Reflectance spectra recorded in air on a nPSiO$_2$ scaffold oxidized at 1000°C for 5 min before and after each coating step with PAH:Rh and PMAA:Rh. c) EOT-EOT$_{nPSiO_2}$ values recorded for as-prepared nPSi scaffolds and oxidized nPSiO$_2$ scaffolds both before and after each coating step with PAH:Rh and PMAA:Rh. The EOT value of as-oxidized nPSiO$_2$ scaffolds (i.e., EOT$_{nPSiO_2}$) is used as reference (n=3 scaffolds for each polyelectrolyte architecture). All data are presented as mean (± s.d).

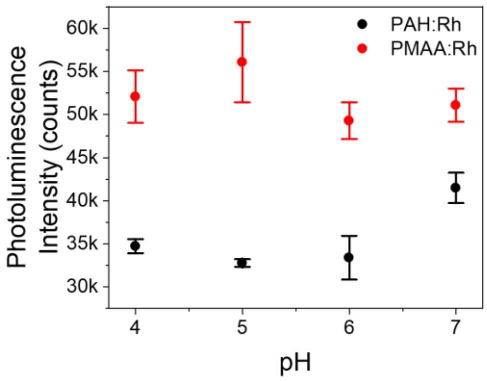

Figure S7. **Intrinsic sensitivity of PAH:Rh and PMAA:Rh to pH.** Photoluminescence intensity at 580 nm of PAH:Rh and PMAA:Rh solutions in the pH range 4 – 7 (n=3 solutions). All data are presented as mean (± s.d).

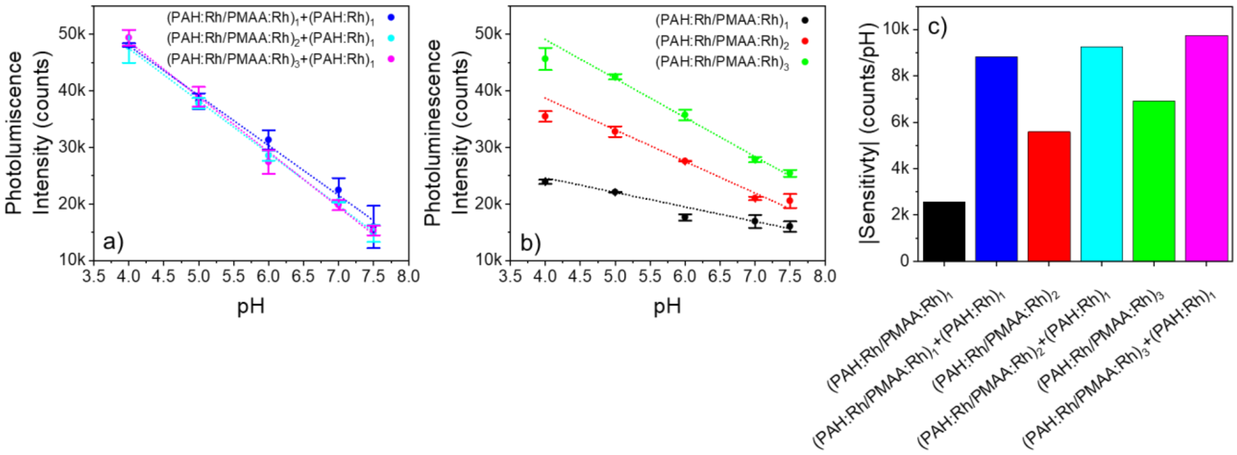

Figure S8. **Effect of the fluorescent polymer stack architecture assembled within the nPSiO2 scaffolds on the pH sensing performance.** a) Calibration curve (Photoluminescence intensity at 580 nm *vs.* pH value) of nPSiO$_2$ scaffolds on silicon chip coated with an odd number of PAH:Rh and PMAA:Rh layers (n=3 pH cycles). b) Calibration curve (photoluminescence intensity at 580 nm *vs.* pH value) of nPSiO$_2$ scaffolds on silicon chip coated with an even number of PAH:Rh and PMAA:Rh layers (n=3 pH cycles). c) Sensitivity to pH of the sensors in a) and b). The assembly buffer was TRIS at pH 8 for all the sensors. Data in a) and b) are presented as mean (± s.d).

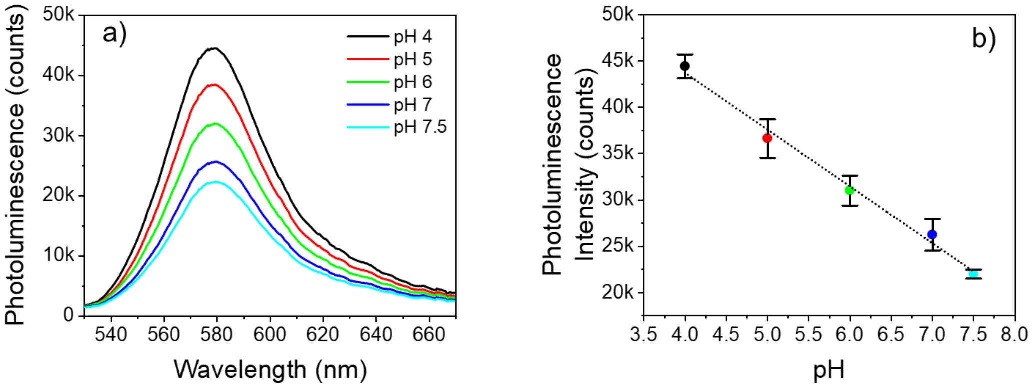

Figure S9. **In-vitro pH sensing with nPSiO$_2$ scaffolds on PLGA foil coated with fluorescent polymer stack.** a) Photoluminescence spectrum of nPSiO$_2$ scaffolds on PLGA foil coated with (PAH:Rh/PMAA:Rh)$_2$+(PAH:Rh)$_1$ measured in PBS solutions with different pH values in the range 4 – 7.5. b) Calibration curve (photoluminescence intensity at 580 nm *vs.* pH value) of sensor in a) (n=3 pH cycles). Data b) are presented as mean (± s.d).

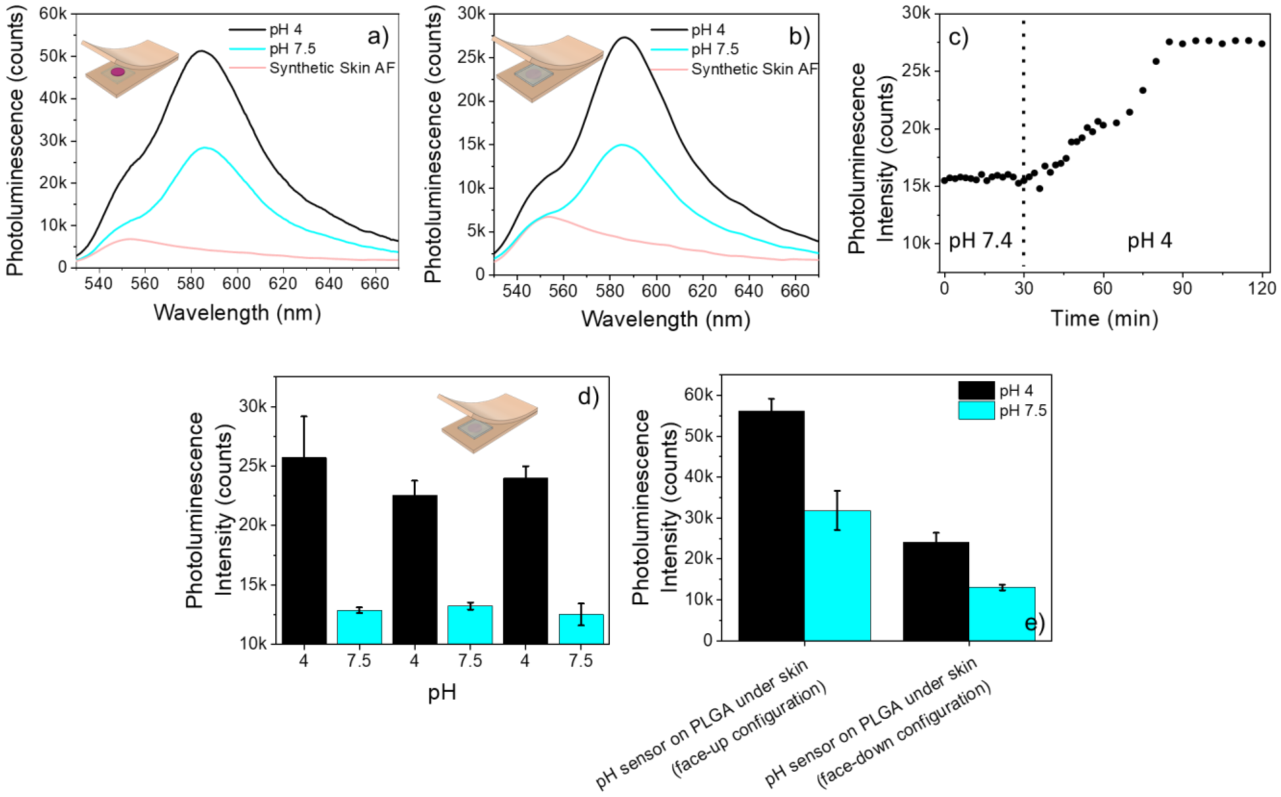

Figure S10. **pH sensing with fluorescent-polymer coated nPSiO$_2$ scaffolds on PLGA foil under synthetic skin.** a, b) Photoluminescence spectra of the pH sensor acquired through synthetic skin conditioned at pH 4 (black) and pH 7.5 (light blue); synthetic skin autofluorescence is also shown. The sensor is placed between two flaps of the skin with the pH-responsive polymer stack face-up (i.e., pointing toward the spectrometer probe) in a) and face-down (i.e., pointing on the opposite side of the spectrometer probe). c) Time-resolved photoluminescence intensity (at 580 nm) of the sensor under synthetic skin in physiological conditions at pH 7.4 and after injection of a PBS solution with

pH 4 around the sensor site. d) Photoluminescence intensity at 580 nm upon alternate injections of PBS solutions with pH of 4 and 7.5, with the sensor sandwiched between two synthetic skin flaps in face-down configuration (n=3 pH sensor for each pH value). e) Comparison between face-up and face-down configurations of the pH sensor within the synthetic skin in different pH conditions (n=3 pH sensor for each configuration). Data d) and e) are presented as mean (± s.d).

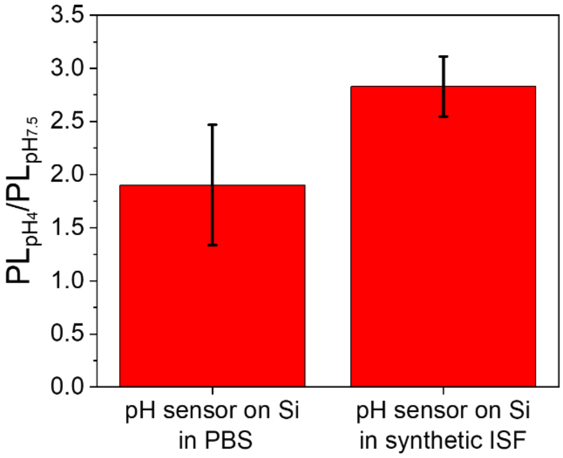

Figure S11. Comparison of pH sensor in PBS solution and synthetic ISF. Photoluminescence intensity ratio between pH 4 and 7.5 measured on the pH sensor on Si in PBS and in synthetic ISF (n=3 samples, 3 full cycles per sample). Data are presented as mean (± s.d.).

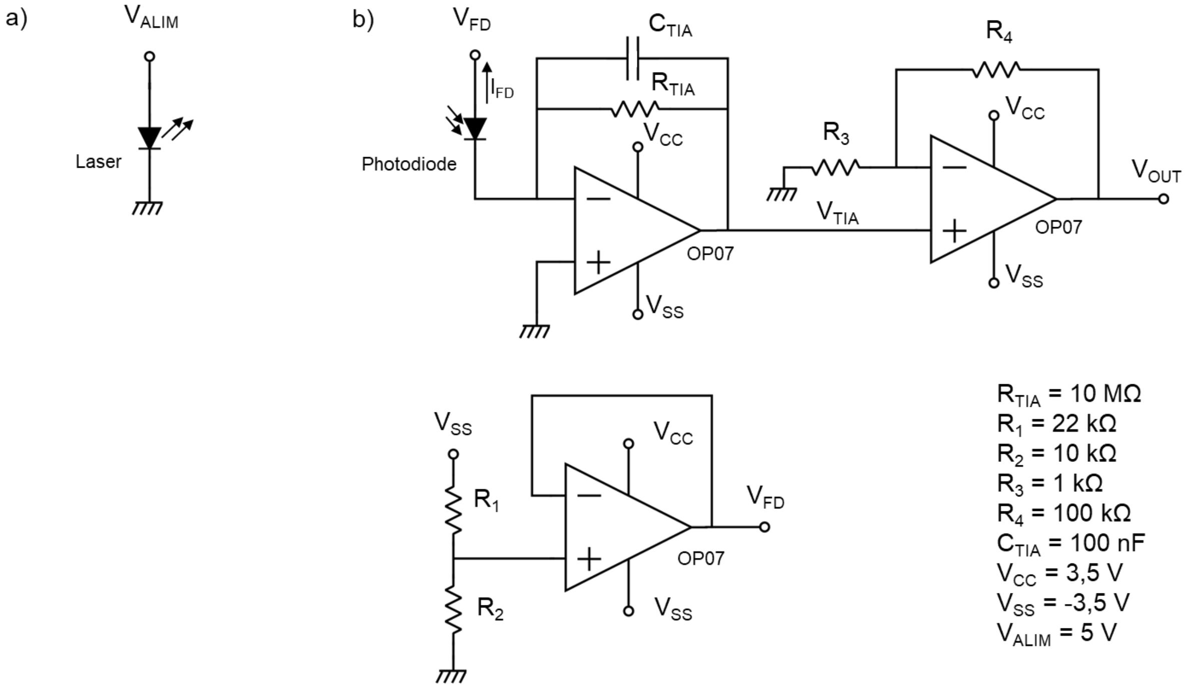

Figure S12. Driving/readout circuit of the laser/photodiode pair. a) Driving circuit of laser. b) Readout circuit: on top, the photocurrent I_{FD} is converted into the voltage V_{TIA} by means of a transimpedance amplifier (TIA) and next amplified by a factor 101 using a non-inverting amplifier, thus obtaining the output voltage V_{OUT}. At the bottom, the photodiode bias voltage V_{FD} is obtained from the voltage source V_{SS} buffering the resistive voltage divider.

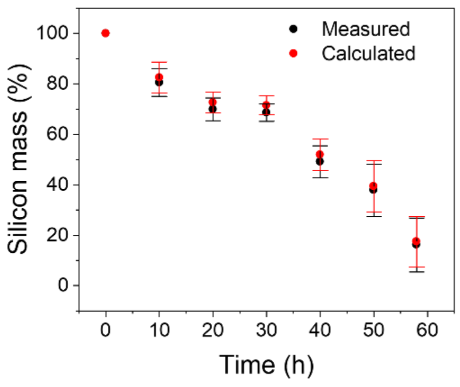

Figure S13. **Simulation of the dissolution kinetics of nPSiO$_2$ scaffolds.** Measured and simulated mass percentage of nPSiO$_2$ scaffolds oxidized at 1000 °C for 5 min at dissolution time in HEPES buffer. The mass percentage of the as-prepared nPSiO$_2$ scaffold (at 0 hour) is used as reference. (n=4 pH cycles for each pH value). Data b) are presented as mean (± s.d).

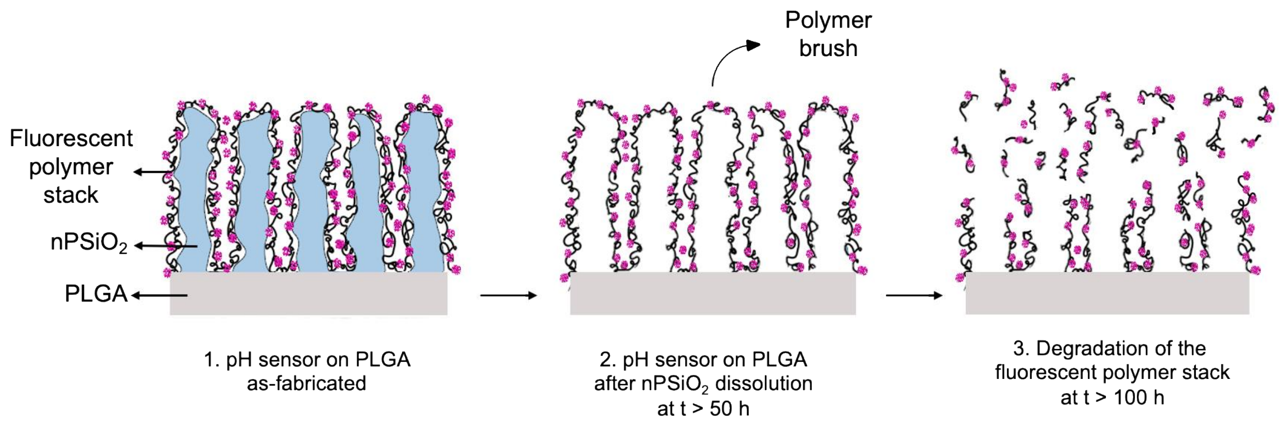

Figure S14. Main dissolution steps of pH sensor on PLGA foil. Sketch of: 1. layer-by-layer conformal coating of the nPSiO$_2$ scaffold with a nanometer-tick fluorescent polymer stack on PLGA foil. 2. pH sensor after nPSiO$_2$ dissolution at t > 50 hours. 3. Degradation of the fluorescent polymer stack at t > 100 hours.

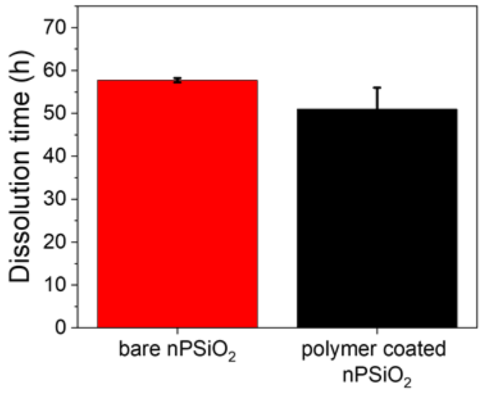

Figure S15. Comparison of dissolution times of bare $nPSiO_2$ and $nPSiO_2$ coated with $(PAH:Rh/PMAA:Rh)_2+(PAH:Rh)_1$ polymer stack (n=3 samples for each configuration). Two-tailed t-student tests (significance level < 0.05) confirmed that the values are statistically equivalent. Data are presented as mean (± s.d).

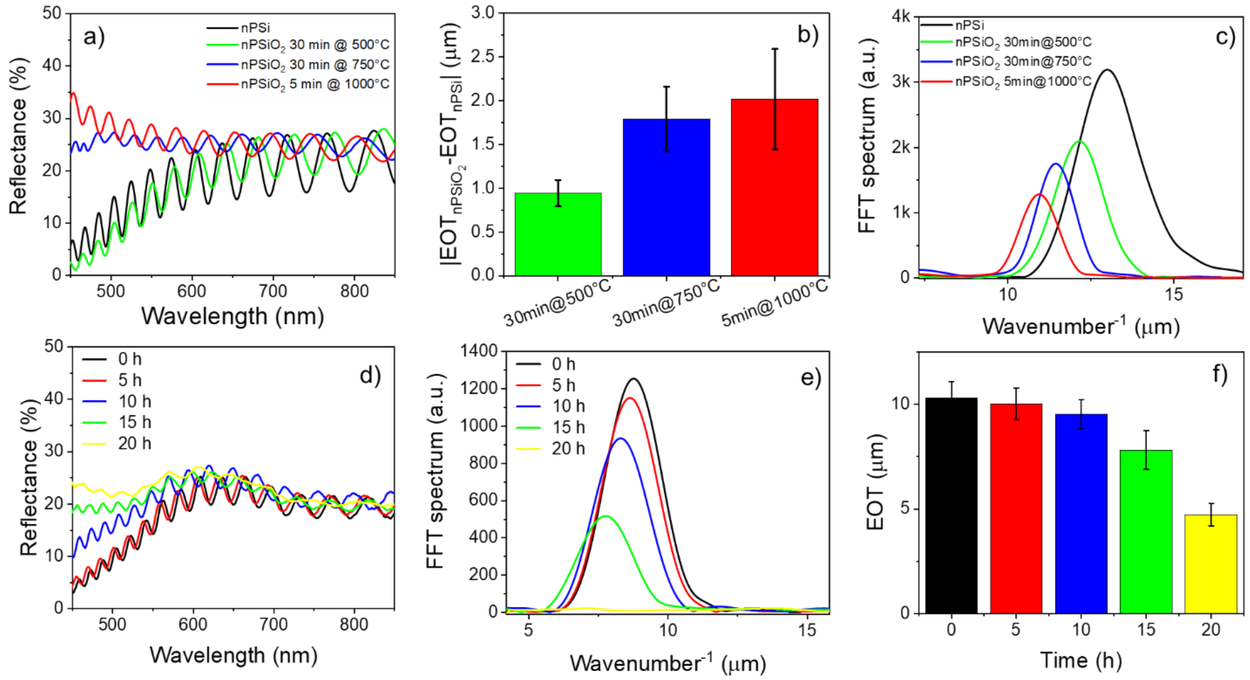

Figure S16. **Optical characterization of $nPSiO_2$ scaffolds during dissolution experiments.** a) Reflectance spectra of $nPSiO_2$ scaffolds oxidized at 1) 500 °C for 30 min, 2) 750 °C for 30 min, 3) 1000 °C for 5 min. b) EOT-EOT_{nPSi} values recorded for as-prepared and oxidized porous scaffolds in a). The EOT value of nPSi (i.e., EOT_{nPSi}) is used as reference (n=4 pH sensor for each oxidation temperature). c) FFT spectrum of as-prepared and oxidized scaffolds in a). The amplitude spectrum shows a monotonic red-shift effect from nPSi to $nPSiO_2$ as the oxidation temperature increases. d) Reflectance spectra of $nPSiO_2$ oxidized at 500 °C for 30 min acquired at different times during dissolution experiments in HEPES. A steady reduction of the fringe amplitude in the reflectance spectrum is evident as the dissolution time increases. e) FFT spectrum of of $nPSiO_2$ scaffolds oxidized at 500 °C for 30 min acquired at different times during dissolution experiments in HEPES. A steady reduction of the amplitude of the FFT spectrum is evident as the dissolution time increases. f) EOT values recorded on $nPSiO_2$ oxidized at 500 °C for 30 min acquired at different

times during dissolution experiments in HEPES (n=3 pH sensor for each dissolution time). Data b) and f) are presented as mean (± s.d).

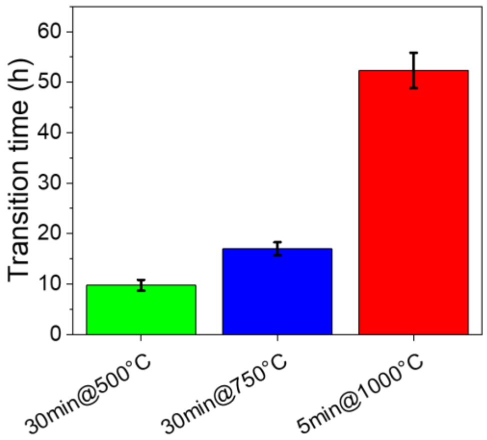

Figure S17. **Transition between oxide and silicon dissolution regimes in nPSiO$_2$ scaffolds.** Transition time between oxide and silicon dissolution regimes in nPSiO$_2$ scaffolds oxidized at 1) 500 °C for 3 min, 2) 750 °C for 30 min, 3) 1000 °C for 5 min (n=4 pH sensor for each oxidation temperature). Data are presented as mean (± s.d).

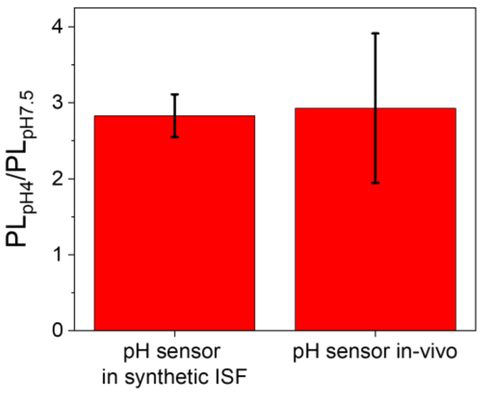

Figure S18. Photoluminescence intensity ratio between pH 4 and 7.5 measured with the pH sensor in vitro in ISF and in vivo (n=3 samples, 3 full cycles per sample). Two-tailed t-student test (significance level < 0.01) confirmed that data were statistically equivalent. Data are presented as mean (± s.d.).

**Animal**	**Total point scale**
Mice sham (*n=5*)	0
Mice with device (*n=6*)	0

Figure S19. Macroscopic evaluation of the implant skin area. After 60 days from implantation of the pH sensor under skin, the back of mice was shaved and the skin surgical area was observed for each mouse and evaluated with a 4-point scale: 0 = intact skin; 1 = swelling, 2 = redness, 3 = eschar.

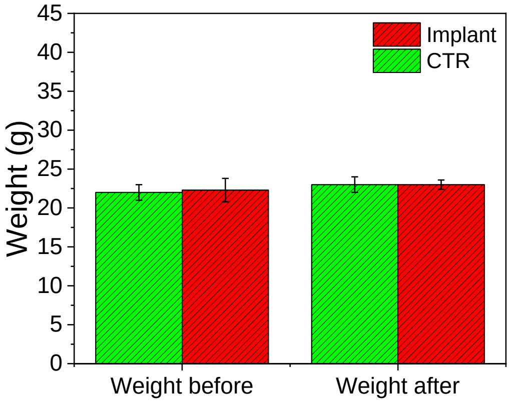

Figure S20. Weight evaluation of mice implanted. Mice (CTR=5, implant=6) were weighted before surgical insertion of device and after two months (last experimental day) (n=5 mice CTR, n = 6 mice with device). Two-tailed t-student test (significance level < 0.01) confirmed that data were statistically equivalent. Data are presented as mean (± s.d.).

References

1. Sailor MJ. *Porous Silicon in Practice: Preparation, Characterization and Applications*. Weinheim, Germany: Wiley-VCH Verlag GmbH & Co. KGaA; 2011. doi:10.1002/9783527641901

2. Mariani S, Robbiano V, Iglio R, et al. Moldless Printing of Silicone Lenses with Embedded Nanostructured Optical Filters. *Adv Funct Mater*. 2020;30(4):1-13. doi:10.1002/adfm.201906836

3. Wan Y, Krueger NA, Ocier CR, Su P, Braun P V., Cunningham BT. Resonant Mode Engineering of Photonic Crystal Sensors Clad with Ultralow Refractive Index Porous Silicon Dioxide. *Adv Opt Mater*. 2017;5(21):1-7. doi:10.1002/adom.201700605

4. Lehmann V. *Electrochemistry of Silicon*. Vol 3.; 2002. doi:10.1002/3527600272

5. Mariani S, Paghi A, La Mattina AA, Debrassi A, Dähne L, Barillaro G. Decoration of Porous Silicon with Gold Nanoparticles via Layer-by-Layer Nanoassembly for Interferometric and

Hybrid Photonic/Plasmonic (Bio)sensing. *ACS Appl Mater Interfaces*. 2019;11(46):43731-43740. doi:10.1021/acsami.9b15737

6. Mariani S, Robbiano V, Strambini LM, et al. Layer-by-layer biofunctionalization of nanostructured porous silicon for high-sensitivity and high-selectivity label-free affinity biosensing. *Nat Commun*. 2018;9(1). doi:10.1038/s41467-018-07723-8
7. Schneider CA, Rasband WS, Eliceiri KW. NIH Image to ImageJ: 25 years of image analysis. *Nat Methods*. 2012;9(7):671-675. doi:10.1038/nmeth.2089
8. Ruminski AM, Barillaro G, Chaffin C, Sailor MJ. Internally referenced remote sensors for HF and Cl2 using reactive porous silicon photonic crystals. *Adv Funct Mater*. 2011;21(8):1511-1525. doi:10.1002/adfm.201002037
9. Astrova E V., Tolmachev VA. Effective refractive index and composition of oxidized porous silicon films. *Mater Sci Eng B Solid-State Mater Adv Technol*. 2000;69:142-148. doi:10.1016/S0921-5107(99)00236-6
10. Earth D, Studies L. *Guide for the Care and Use of Laboratory Animals*. National Academies Press; 2011. doi:10.17226/12910
11. CEE Council 89/609; Italian D.L. 26/2014, No. n° 979/2020-PR
